# Dissecting the Origin of Heterogeneity in Uterine and Ovarian Carcinosarcomas

**DOI:** 10.1158/2767-9764.CRC-22-0520

**Published:** 2023-05-10

**Authors:** Anne-Sophie Sertier, Anthony Ferrari, Roxane M. Pommier, Isabelle Treilleux, Sandrine Boyault, Mojgan Devouassoux-Shisheboran, Janice Kielbassa, Emilie Thomas, Laurie Tonon, Vincent Le Texier, Amandine Charreton, Anne-Pierre Morel, Anne Floquet, Florence Joly, Dominique Berton-Rigaud, Gwenaël Ferron, Laurent Arnould, Sabrina Croce, Guillaume Bataillon, Pierre Saintigny, Eliane Mery-Lamarche, Christine Sagan, Aruni P. Senaratne, Ivo G. Gut, Fabien Calvo, Alain Viari, Maria Ouzounova, Isabelle Ray-Coquard, Alain Puisieux

**Affiliations:** 1 Synergie Lyon Cancer, Plateforme de bioinformatique Gilles Thomas, Centre Léon Bérard, Lyon, France.; 2 Centre Léon Bérard, Lyon, France.; 3 Department of Pathology, Centre Léon Bérard, Lyon, France.; 4 Department of Pathology, Hospices Civils de Lyon, Lyon, France.; 5 Université de Lyon, Université Claude Bernard Lyon 1, INSERM 1052, CNRS 5286 Centre Léon Bérard, Cancer Research Center of Lyon, Lyon, France.; 6 Institut Bergonié Comprehensive Cancer Centre, Bordeaux, France.; 7 Centre François Baclesse, Caen, France.; 8 Institut de Cancérologie de l'Ouest René-Gauducheau, Saint-Herblain, France.; 9 Institut Claudius-Regaud, IUCT Oncopole, Toulouse, France.; 10 Department of Pathology, Centre Georges François Leclerc, Comprehensive Cancer Centre, Dijon, France.; 11 Department of Biopathology, Institut Bergonié Comprehensive Cancer Centre, Bordeaux, France.; 12 Service de Pathologie, Institut Curie, Paris, France.; 13 Department of Translational Medicine, Centre Léon Bérard, Lyon, France.; 14 Department of Medical Oncology, Centre Léon Bérard, Lyon, France.; 15 Institut Curie, PSL Research University, Paris, France.; 16 CNAG-CRG, Centre for Genomic Regulation (CRG), Barcelona Institute of Science and Technology (BIST), Carrer Baldiri i Reixac 4, Barcelona, Spain.; 17 Universitat Pompeu Fabra (UPF), Barcelona, Spain.; 18 Centre de Recherche des Cordeliers, Université de Paris-Cité, Paris France.; 19 Chemical Biology of Cancer Laboratory, CNRS UMR 3666, INSERM U1143, Paris, France.

## Abstract

Gynecologic carcinosarcomas (CS) are biphasic neoplasms composed of carcinomatous (C) and sarcomatous (S) malignant components. Because of their rarity and histologic complexity, genetic and functional studies on CS are scarce and the mechanisms of initiation and development remain largely unknown.

Whole-genome analysis of the C and S components reveals shared genomic alterations, thus emphasizing the clonal evolution of CS. Reconstructions of the evolutionary history of each tumor further reveal that C and S samples are composed of both ancestral cell populations and component-specific subclones, supporting a common origin followed by distinct evolutionary trajectories. However, while we do not find any recurrent genomic features associated with phenotypic divergence, transcriptomic and methylome analyses identify a common mechanism across the cohort, the epithelial-to-mesenchymal transition (EMT), suggesting a role for nongenetic factors in inflicting changes to cellular fate. Altogether, these data accredit the hypothesis that CS tumors are driven by both clonal evolution and transcriptomic reprogramming, essential for susceptibility to transdifferentiation upon encountering environmental cues, thus linking CS heterogeneity to genetic, transcriptomic, and epigenetic influences.

**Significance:**

We have provided a detailed characterization of the genomic landscape of CS and identified EMT as a common mechanism associated with phenotypic divergence, linking CS heterogeneity to genetic, transcriptomic, and epigenetic influences.

## Introduction

Endometrial carcinosarcomas (CS) represent approximately 2% to 5% of uterine malignancies, but cause around 16% of all deaths due to malignancies of the uterine corpus (1). Although endometrial CS share similar risk factors with endometrial carcinomas such as obesity, nulliparity, smoking, and exogenous estrogen use, these malignancies are diagnosed at more advanced stages and accompany significantly worse chances of survival than other high-grade endometrial carcinomas (1). Similarly, patients with ovarian CS often present with advanced stage disease and symptoms similar to those of epithelial ovarian cancer. Together, endometrial and ovarian CS are rare aggressive diseases that are characterized by a biphasic histology. This heterogeneous pathology underlies the presence of two phenotypic components: a carcinomatous (C) component displaying epithelial characteristics and a sarcomatous (S) component associated with mesenchymal features. The fraction of C and S tissue in each CS tumor impacts disease prognosis, degree of metastasis, likelihood of disease recurrence, and survival rate. Notably, a poorer prognosis is associated with highly heterogeneous tumors over purely carcinomatous ones (2, 3). Thus, it is important to understand the origin of CS heterogeneity and integrate these data within the current molecular subgroups.

Four molecular groups have been recently defined for endometrial carcinomas: the hypermutated (mismatch repair deficiency), the ultramutated (*POLE* mutated), the copy-number low (CN-low), and the copy-number high (CN-high) groups. These groups not only display different molecular alterations but also present varied prognoses: patients from the ultramutated group are associated with the best prognosis, whereas patients in the CN-high group have the highest risk of recurrence (4). The same four molecular groups can also stratify endometrial CS, with most belonging to the CN-high serous-like molecular subtype, characterized by frequent *TP53* mutation and amplification of oncogenes such as *CCNE1* and *c-MYC* (3). To date however, there is an unmet need for a comprehensive characterization of CS heterogeneity. The vast majority of previous reports have explored through multi-omics approaches the bulk of CS tumors. While these studies have provided important insights into CS tumorigenesis, they have failed to discriminate between the two histologic components. Furthermore, recent analysis of microdissected samples of the two components has relied on DNA methylation patterns or whole-exome sequencing (WES) to investigate the molecular landscape of CS (3, 5). However, WES approaches prevent the identification of genomic rearrangements or an accurate detection of the mutational processes and limit the identification of mutations to only the coding regions of the genome. Therefore, despite an increasing body of evidence, the pathogenesis of CS remains largely unknown.

Here, we report a comprehensive analysis of macrodissected samples of C and S components through whole-genome sequencing (WGS), DNA methylation profiling, and RNA sequencing (RNA-seq). Our genomic data reveal new insights into the mutational signatures underlying the tumorigenesis of CS and corroborate previous studies emphasizing the clonal evolution of CS stemming from a common origin of the two components. Importantly, the nature and frequency of these mutational processes further allowed us to assess the contribution of genetic factors to CS heterogeneity. Together with methylation and gene expression data, the heterogeneity of CS tumors is revealed to be orchestrated by genetic, epigenetic, and transcriptomic cues, and suggest a role of nongenetic reprogramming in driving the CS phenotypic switch.

## Materials and Methods

### Patients and Ethical Approvals

A total of 62 female patients diagnosed with gynecologic CS (GCS) cancer were recruited through eight French centers: Bergonié Institute (Bordeaux), Center George François Leclerc Center (CGFL, Dijon), Lyon Croix Rousse Hospital, CHU of Saint Etienne, François Baclesse Center (Caen), West Cancerology Institute, René Gauducheau (Nantes), Institut Curie (Paris), and Léon Bérard Center (CLB, Lyon; Supplementary Fig. S1). The trial was sponsored by the French National Cancer Institute (INCa) and approved by the central ethical committee on May 15, 2006. It was done in compliance with the principles of Good Clinical Practice and the Declaration of Helsinki and registered at ClinicalTrials.gov, number NCT00381901. Patients were eligible if they were over 18 years of age with histologically confirmed gynecologic CS cancer and had provided signed informed consent.

### Collection and Review of Samples

For each patient, tumor tissue as well as matched blood samples were collected within the French rare gynecologic cancer network (TMRG; www.ovaire-rare.org). Tumors were snap-frozen in liquid nitrogen upon surgical removal after pathologist's review and were stored in the corresponding hospital's biological resources center. All tumor formalin-fixed paraffin-embedded (FFPE) slides were reviewed by a pathologist. Corresponding pathologic, clinical, and follow-up data were obtained from the collecting centers.

### Sample DNA and RNA Extraction

Macrodissection was performed to extract C and S samples. DNA and RNA were then extracted from both samples. Total genomic DNA was extracted with phenol-chloroform after proteinase K digestion, followed by the precipitation of nucleic acids in ethanol. DNA was quantified using Nanodrop spectrophotometer ND-1000 (Thermo Fisher Scientific) and Qubit HS DNA assay (Invitrogen). RNA was also extracted using the miRNeasy miniKit (Qiagen) in accordance with the manufacturer's protocol. RNA was quantified using Nanodrop spectrophotometer ND-1000 and the purity and integrity were assessed by the Agilent 2100 Bioanalyzer and RNA 6000 Nano Labchip Kit (Agilent Biotechnologies). All matched peripheral blood samples have been centralized and then extracted using the salting out method with a Qiagen Autopure LS in the Fondation Jean Dausset CEPH laboratory. To confirm a match between tumoral and blood DNA issued from the same patient, the AmpFLSTR Identifiler PCR Amplification Kit (Life Technologies) has been used.

### Selection of CS Samples

A total of 62 GCS tumors (and matched blood samples) were processed (Supplementary Fig. S1). Special attention was then paid to the selection of a high-quality subset of the tumor samples for further analysis. The proportion of tumor cells was estimated from frozen tumor sections by pathologists and only those estimated to have at least 50% tumor cells were retained. All DNA and RNA samples were subjected to quality controls (RNA integrity number ≥7; DNA integrity checked on agarose gel) leaving a subset of 40 samples corresponding to 20 patients. The DNA of these tumor samples was hybridized on Illumina Cytoscan arrays to establish the genomic profile of each tumor. These genomic profiles were used to obtain another estimation of tumor purity (see SNP array processing section below for details). A very low estimate of purity, less than 20%, caused the sample to be discarded. At the end of this process, both samples of 15 tumors met the required quality criteria, all of which were then subjected to WGS, RNA-seq, and methylation array analysis.

### WGS and Processing

WGS was performed on 15 sets of three samples composed of two tumoral DNA samples and a matched normal DNA sample from the same individual. Illumina HiSeq2000/HiSeq2500 genome analyzers and Illumina paired-end sequencing protocols were used for all samples, read lengths were 2 × 126 bp. Tumor DNA and normal DNA were sequenced to 77-fold and 52-fold median coverage, respectively. Paired-end reads were aligned to the human genome (GRCh37) using the Burrows-Wheeler aligner (6). Alignments were refined using GATK v3.1-1 (7) and Picard v1.107 (http://broadinstitute.github.io/picard/) software suites. Duplicates were removed from the sample BAM files for further analysis. Raw and mapped sequences from all produced HiSeq lanes were checked using in-house pipelines that collect a set of metrics reflecting the overall quality of the sequencing data. All lanes showed a median per-cycle base quality score higher than Q30 (phred-score). More than 90% of reads were uniquely mapped for all lanes.

### Somatic Single-nucleotide Variation and Small indel Call

Somatic single-nucleotide variations (SNV) were called using MuTect v1.1.15 part of the GATK3 suite. To improve performance, data from dbSNP Build 132 and COSMIC v65 (http://www.sanger.ac.uk/genetics/CGP/cosmic/) were supplied as parameters to MuTect. Moreover, a panel of normal genomes generated on the same sequencing technology was used to dismiss systematic sequencing errors and/or low-frequency polymorphisms. SNVs that passed all these filters were then annotated using the Variant Effect Predictor (VEP) toolv75 (8). Small insertions/deletions (indel) were called with Mutect2, v3.5-0-g36282e4; the same post-processing filters used to validate SNV was applied.

### Somatic Copy-number Alteration Call

Somatic copy-number alterations (CNA) were called from WGS data using an in-house pipeline (https://github.com/aviari/wginr; refs. 9, 10) that consists of three main steps. First, the dependency between GC content and raw read count is modeled using a generalized additive smoothing model with two nested windows to catch short- and long-distance dependencies. In a second step, we collect heterozygous positions in the matched normal sample and GC-corrected read counts (RC) and allelic frequencies (AF) at these positions are used to estimate the mean tumor ploidy and its contamination by normal tissue. This ploidy model is then used to infer the theoretical absolute copy-number levels in the tumor sample. In the third step, a simultaneous segmentation of RC and AF signals is performed using a bivariate Hidden Markov Model to generate an absolute copy number and a genotype estimate for each segment.

The fraction of genome altered (FGA) is computed as the proportion of the genome with a copy number different from the tumor modal ploidy. A CNA breakpoint is defined as a change of CN between two adjacent segments.

### Somatic Structural Variant Call

Somatic structural variants (SV) were identified using an in-house tool (https://github.com/anso-sertier/crisscross; refs. 9, 10) that uses WGS data and two complementary signals from the read alignments: (i) discordant pair mapping (wrong read orientation or incorrect insert-size); and (ii) soft-clipping (unmapped first or last bases of read) that allows to resolve SV breakpoints at the bp. A cluster of discordant pairs and one or two clusters of soft-clipped reads defined an SV candidate: the discordant pairs cluster defined two associated regions, possibly on different chromosomes and the soft-clipped reads cluster(s), located in these regions, pinpointed the potential SV breakpoint positions. We further checked that the soft-clipped bases at each SV breakpoint were correctly aligned in the neighborhood of the associated region. SV events were then classified as germline or somatic depending on their occurrence in the matched normal sample. SV events were classified into four classes according to discordant pair orientations: deletion, inversion, duplication, and interchromosomal translocation.

SV breakpoints were compared with gene position to detect so-called “broken genes”; broken genes were filtered according to the number of reads supporting the SV breakpoints (>15 reads).

### CNA Analysis: Large-scale State Transition

Large-scale state transitions (LST) were defined as chromosomal breaks between adjacent regions of at least 10 Mb each. As described previously, the number of LST in the tumor genome was estimated for each chromosome arm independently (not accounting for breaks at centromeric or unmappable regions) after filtering and smoothing of all variations less than 3 Mb. High number of LST (>20) is a surrogate indicator of BRCAness status (11).

### Tandem Duplicator Phenotype Analysis

To detect tandem duplicator phenotypes (TDP) in tumor samples, two criteria were checked: (i) a TDP score was computed as described in Menghi and colleagues: a positive score indicated TDP, (ii) TDP tumors display a high number of tandem duplications with a spanning size of around 10 kb (12).

### Mutational Signature Landscape

An in-house analysis pipeline was used to decipher and analyze mutational signatures (https://github.com/EmilieT/mutcraft). First the non-negative matrix factorization (NMF) method described by Alexandrov and colleagues (13) was used to *de novo* decipher mutational signatures using all tumor samples. The method has been implemented in R using the NMF package available on CRAN.

The decomposition was tested for a number of signatures ranging from 2 to 10. To test the robustness of the solution, each decomposition was bootstrapped 1,000 times by applying a Monte Carlo resampling on the starting matrix. To determine the number of optimal solutions, the stability of the decomposition was ensured by using the metrics included in the NMF package, by clustering the coefficients of the 1,000 signatures to ensure their similarities, and by ensuring that the weight of the residuals is minimal. The optimal decomposition with respect to these criteria was carried out in four signatures. Each resulting signature corresponds to the average of the 1,000 bootstraps.

The four signatures obtained were then compared with those of COSMIC (https://cancer.sanger.ac.uk/cosmic/signatures_v2.tt). This comparison was done using cosine similarity. The clustering was made using 1-cosine as distance and Ward's linkage.

### Kataegis Detection

APOBEC-related kataegis was called if four criteria were met: (i) detection of local hypermutation events based on k-nearest neighbor methodology, (ii) mutation spectrum of SNV constituting the kataegis was composed of TC>G, TC>T, or TC>A substitutions, (iii) mutations display similar variant allele frequencies (VAF) and were assigned to the same subclonal population (as they are supposed to occur during the same event), and (iv) the local hypermutation event was found near a genomic rearrangement.

### Microsatellite Instability Score

A next-generation sequencing–based microsatellite instability (MSI) score was computed using MSIsensor-v0.6 (14) for each tumor sample. This tool computes both length distributions of microsatellites from the tumor and its matched normal data and then compare these distributions to infer putative instability of each site. MSIsensor computes the proportion of unstable sites among a given list of sites. We used the list of 2,932 most unstable sites published by Salipante and colleagues (15). A tumor was considered as MSI if the fraction of unstable sites was >20%.

### Homologous Recombination Deficiency Status

Homologous recombination deficiency (HRD) status was designed as follow: high status was attributed to samples presenting either a gene alteration in *BRCA1/2* genes or a LST number >16, low status was assigned to samples without any *BRCA1/2* gene alteration and presenting a LST number <6. All remaining samples were labeled as intermediary.

### Clonal Evolution

#### Inference

To build the subclonal populations phylogeny, a two-step strategy combining both CNA and point mutations signals was used: (i) a first step consisting of the identification of CNA subclonal populations in each sample with the Battenberg algorithm (16), and (ii) a second step relying on the incorporation of allelic frequencies of somatic mutations with phyloWGS algorithm (17).

#### Genome Doubling Timing

For tumors with modal ploidy greater than three, we investigated the timing of genome doubling events by analyzing mutations occurring on genomic segments with balanced copy number of four (A2B2 genotype). Mutations arising before genome doubling have theoretical VAF (without accounting for normal contamination) of 0.5, whereas mutations occurring after genome doubling (on one allele among four) have a lower theoretical VAF of 0.25. Mutations were thus classified as before or after a genome doubling event. By analyzing the composition of these sets of mutations in subclonal populations, we could assign the population in which the genome doubling event was most probable to have occurred.

#### Mutational Signature Landscape

Mutational signature proportion was estimated for each set of mutations assigned to a subclonal population. Mutations were assigned to the most probable mutational signature (from NMF decomposition output) allowing us to estimate the contribution of each of the four signatures identified in the cohort.

### RNA-seq: Expression and Fusion Transcript Detection

RNA-seq was performed on each of 15 paired tumor samples. RNA was sequenced on Illumina HiSeq2000/HiSeq2500 genome analyzers and Illumina paired-end sequencing protocols (2 × 76 bp) were used for all samples. Paired-end reads were aligned to the human genome (GRCh37) and quantified using STAR algorithm (18). Transcript per million (TPM) normalization was applied to raw gene expression data. Quality controls were done with FastQC (v0.11.5), all lanes passed default thresholds of sequencing quality.

Candidate fusions were called with STAR-fusion and filters suggested by the authors were applied (19). Next, each fusion retained was compared with SVs to detect genomic events at the origin of the fusion.

### Methylome Analysis

High-throughput DNA methylation analysis was performed on the Illumina Infinium HumanMethylation 450 BeadChip Arrays. Quality control, filtering, and normalization were performed with Rnbeads (20). Beta values were computed for each gene promoter: a beta value <0.3 pointed out hypomethylated promoters and a beta value >0.7 indicated hypermethylated promoters.

### Differential Analysis and Pathway Enrichment

We performed paired differential analysis for both expression and methylation data to catch differences between C and S components of the five selected tumors.

Differential expression analysis was done with DESeq2 algorithm while differential methylation analysis was performed on gene promoter regions using RnBeads.

Significantly differentially expressed genes, as well differentially methylated gene promoters were used to perform gene set overrepresentation analysis on MSigDB Hallmark and C2 collections (21–23).

### Pathway Scores

Epithelial-to-mesenchymal transition (EMT) and stemness-related pathways (Hallmark EMT, Lim mammary stem cell, and Boquest stem cell; refs. 22, 24, 25) were projected by using the single-sample extension of GSEA (ssGSEA; ref. 26). TPM-normalized gene expression values for each single sample were ranked, and an enrichment score was produced using the empirical cumulative distribution functions of the genes in the signature and in the remaining genes. EMT scores (Tan_Thiery EMT) were generated according to the methodology described in Tan and colleagues (27).

### Data Availability

Sequencing data that support the findings of this study have been deposited in the European Genome-Phenome archive. The raw data for WGS, RNA-seq, and methylation array are available under study accession EGAS00001002271 (https://ega-archive.org/studies/EGAS00001002271). All scripts and pipelines are available upon request.

## Results

### Characterization of Tumor Samples and Analytic Approach

**FIGURE 1:**
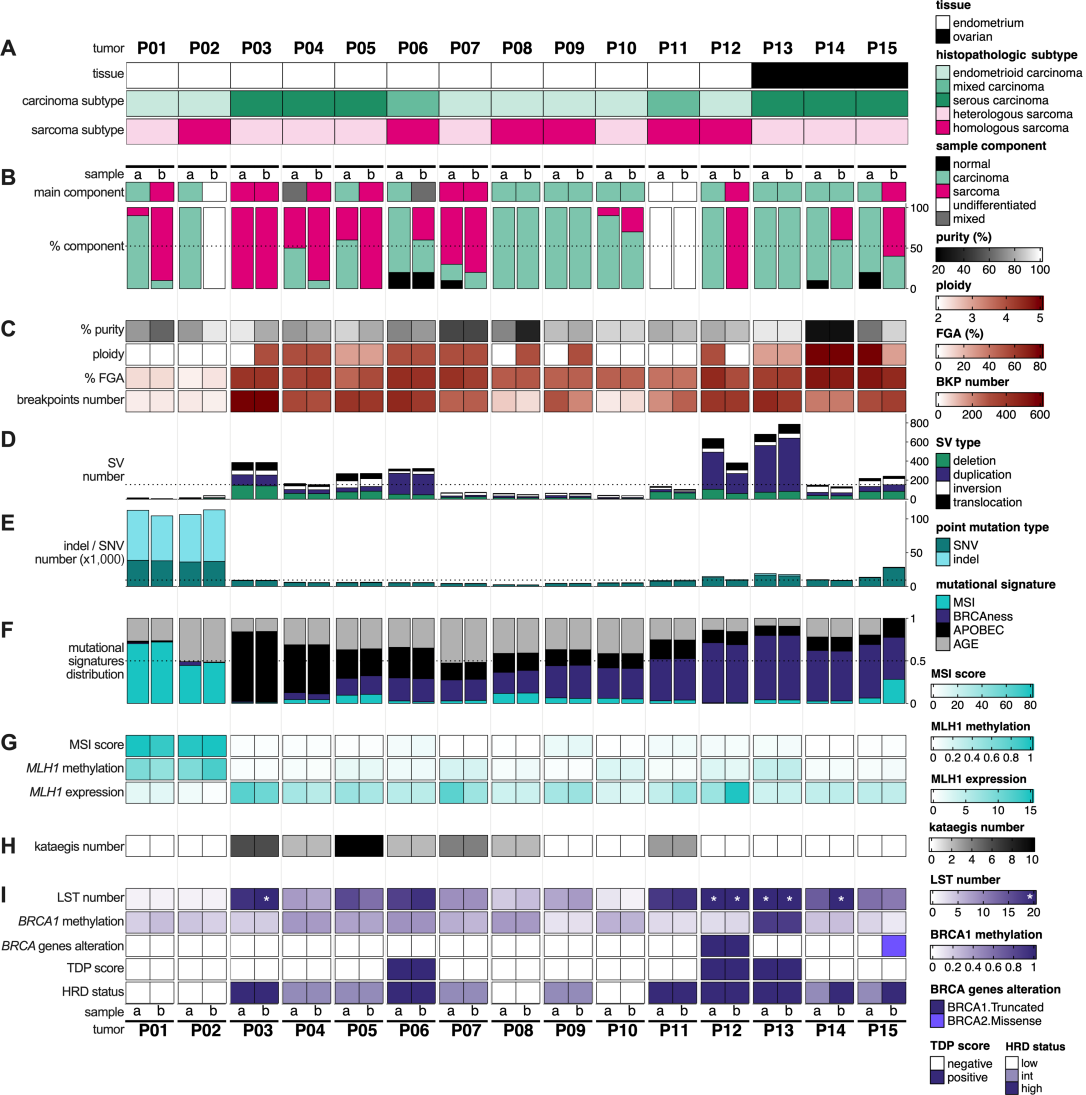
Genomic landscape of CS. **A,** Tissue origin and histological subtypes for each tumor (from P01 to P15). **B,** Main component and detailed percentage content for both samples (a and b) derived from the same tumor. **C,** CNA analysis: tumor purity (%), ploidy, FGA, and CNA breakpoints number. **D,** SV number, classified as deletion, duplication, inversion, and interchromosomal translocation. **E,** SNV and small indel number. **F,** Proportion of each of four mutational signatures deciphered in the whole cohort. **G,** MSI phenotype: MSI score (representing the percentage of unstable microsatellite loci), *MLH1* promoter methylation (beta-value), and mRNA expression level (TPM – transcripts per million). **H,** Kataegis number illustrating APOBEC error-prone DNA repair process. **I,** BRCAness phenotype: number of LST, (white stars highlight samples with LST number ≥20), *BRCA1* promoter methylation level (beta-value), *BRCA1/*2 genes alterations and TDP score, collectively summarized by the HRD status track.

We conducted a French multi-center recruitment to compose a cohort of gynecologic CS comprising 15 paired tumor samples: 12 endometrial and three ovarian CS. The histologic characteristics of the whole cohort are heterogeneous, in accordance with the intrinsic variability of the disease: ovarian CS are composed of a mixture of serous C and heterologous S components, whereas endometrial CS display much more diverse histologic features, with either endometrioid, serous, or mixed carcinomas associated with both heterologous or homologous sarcoma subtypes (Fig. 1A; Supplementary Fig. S1). Pathologist review of FFPE tissue slides delineated specimens into carcinoma (C), sarcoma (S), or undifferentiated (U) phenotype. Macrodissection of fresh-frozen CS samples further allowed the separation of independent tissue regions for each of the 15 selected tumors: (i) five tumors with two distinct regions that could be efficiently separated (C/S or C/U), (ii) eight tumors comprising two regions of the same phenotype (C/C, S/S, or U/U), and (iii) two tumors for which at least one of the two regions consist of intricate C and S components, thereafter defined as “mixed” (M) component (C/M, M/S; Fig. 1B). This sample selection strategy allowed subsequent investigation of the genomic, transcriptomic, and DNA methylomic discrepancies between the C and S components.

### Genomic Rearrangements in CS Tumors are Heterogeneous and Recapitulate the Whole Molecular Spectrum of Uterine and Ovarian Carcinomas

Tumor purity and ploidy were inferred by WGS analysis of CNAs. CS samples showed a low rate of normal cell contamination while mean tumor ploidy was highly variable (from 2 to 5) among patients or within two samples originating from the same tumor (P12; Fig. 1C). In line with ploidy results, profiles of CNA and SV were highly heterogeneous. FGA ranged from 1% to 80% with a median of 56%, while breakpoint numbers varied from 5 to 824 with a median of 317. SV per sample ranged from 6 to 786 (median of 141) and exhibited patterns associated with different proportions of deletion, duplication, inversion, and interchromosomal translocation (Fig. 1C and D).

Both SV and CNA analyses revealed that CS present a substantial heterogeneity in terms of their genomic landscape, with samples displaying either paucity (P01, P02, P10), plenty (P12, P13), or an intermediary phenotype of genomic rearrangements (Fig. 1C and D; Supplementary Fig. S2). As anticipated, serous carcinomatous tumors presented a higher rate of genomic rearrangements (median of CNA breakpoints: 400, SV: 255) compared with endometrioid carcinomatous tumors (CNA: 137, SV: 55, Mann–Whitney *U* test: CNA breakpoints: *P* = 6.92e^−3^, SV: *P* = 7.51e^−4^), as similarly observed in other cohorts (Fig. 1A, C, and D; ref. 28).

### CS Tumorigenesis is Driven by Four Main Mutational Processes

Point mutation analysis revealed an extended heterogeneity in SNVs and small indel, ranging respectively from 2,107 to 38,684 (median: 7,870) and from 216 to 76,293 (median: 651; Fig. 1E). Ovarian samples exhibited a higher median mutation rate (median: 15,628 SNV and indel) than endometrial samples (median: 6,038 SNV and indel). To decipher the underlying mutational processes, *de novo* deconvolution of mutation signatures was carried out. Optimal decomposition was obtained using four signatures. Hierarchical clustering was then used to assign these signatures to known COSMIC mutational processes and thus identified MSI, APOBEC, BRCAness, and the ubiquitous age-related signatures (Fig. 1F; Supplementary Fig. S3; ref. 13). Interestingly, independent of their phenotype (C, S, or U), across the cohort, both samples from the same tumor consistently presented identical signatures in equivalent proportions.

MSI was identified as the main mutational process in two tumors (P01 and P02), supported by their somatic hypermutation status (>100,000 point mutations per sample), low genomic instability (FGA < 10%, breakpoints < 25, and SV < 40), and *MLH1* promoter methylation being inversely correlated to *MLH1* gene expression (Fig. 1C–G). Furthermore, the analysis of known unstable microsatellite loci from WGS data (MSI score) endorsed these results (Fig. 1G; refs. 14, 15). The second key mutational process identified was related to APOBEC activity. Whole-genome detection of kataegis patterns (local hypermutation events) across the cohort corroborated this mutational signature (Fig. 1F and H; Supplementary Fig. S4; ref. 29). Finally, BRCAness-related mutational process was substantiated by alteration of either the *BRCA1* or *BRCA2* gene or *BRCA1* promoter hypermethylation status. In addition, whole-genome detection of a high number (≥20) of LST and TDP further strengthened the BRCAness phenotype (Fig. 1F and I; Supplementary Fig. S5; refs. 11, 12). Remarkably, all three ovarian CS samples mainly exhibited the BRCAness-associated signature.

Altogether, whole-genome analysis revealed that CS tumorigenesis is mainly orchestrated by three genomic mechanisms: MSI, APOBEC-related DNA repair, or BRCAness-related HRD. In particular, our results highlighted the role of APOBEC-related and BRCAness mechanisms in driving genomic instability in CS.

### Frequently Altered Genes in CS Tumors

Analysis of recurrent gene alterations was performed using genes previously attributed to one of the 10 oncogenic signaling pathways described by The Cancer Genome Atlas (TCGA; cell cycle, HIPPO, MYC, NOTCH, NRF2, PI3K/AKT, RTK, RAS, TGFβ signaling, P53, and β-catenin/WNT; ref. 30). In addition, a manually curated list of chromatin remodeling genes (CRG) has been included in the analysis, because chromatin remodeling pathways have been suggested to be involved in CS tumorigenesis (Supplementary Table S1; refs. 31, 32).

**FIGURE 2:**
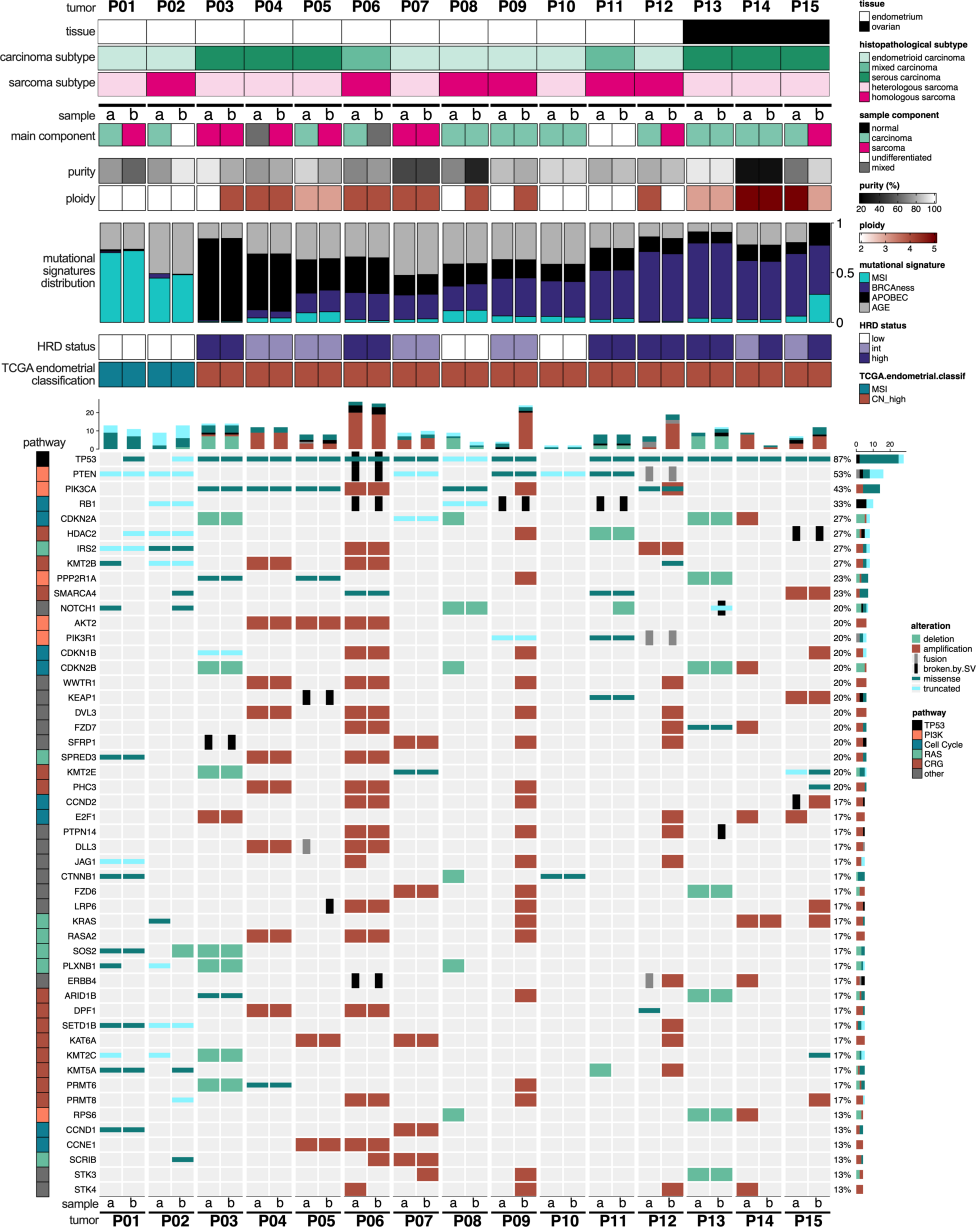
Key gene alterations in uterine and ovarian CS. Oncoprint of alterations identified in the 50 most altered genes from TCGA cancer pathways and custom lists of CRG. The type of genomic alteration (deletion, amplification, fusion, broken by SV, missense, truncated) is described in the legend. Number and proportions of alteration types are summarized by gene (horizontal bars on right) and by sample (vertical bars on top). The samples are classified into MSI and CN-high according to TCGA endometrial carcinoma classification.

Among these 472 selected cancer-related genes, eight were found altered in more than 25% of the cohort, associated to the following pathways: TP53, PI3K/AKT, cell cycle, CRG, and RAS (Fig. 2). Analysis of recurrent gene alterations confirmed the large prevalence of a mutated TP53 pathway among the cohort (*TP53*: 87%, *MDM2*: 10%, *MDM4*: 10%; refs. 3, 32; Supplementary Fig. S6). Likewise, PI3K pathway was found to be largely altered in CS (*PTEN*: 53%, *PIK3CA*: 43%, *PPP2R1A*: 23%), together with genes implicated in cell-cycle regulation (*RB1*: 33%, *CDKN2A*: 27%, *CDKN1B*: 20%), chromatin remodeling (*HDAC2*: 27%, *KMT2B*: 27%, *SMARCA4*: 23%), and RAS pathway (*IRS2*: 27%, *SPRED3*: 20%, *KRAS*: 17%; Fig. 2; Supplementary Fig. S6–S8).

### Molecular Classification of CS Tumors

TCGA-based classification has previously demonstrated the existence of four molecular subtypes of CS distinguished by genomic features (similar to those observed in endometrial carcinoma; refs. 3, 33): *POLE*-mutated, MSI, copy CN-high, and CN-low. These subtypes have been linked to DNA repair deficiencies, clinical and histopathologic features, and therapy outcomes (3). Accordingly, we classified the tumors into these four subtypes (Fig. 2; Supplementary Fig. S9; ref. 28). The two tumors previously assigned to the MSI phenotype (P01 and P02) were coherently attributed to TCGA MSI subtype. The 13 remaining tumors of the cohort were all classified as CN-high according to the previously described classification (ref. 33; Fig. 1; Supplementary Fig. S2). Our study did not enclose any tumors belonging to the *POLE*-mutated or CN-low subtypes, most likely due to the size of the cohort. This result is coherent with a frequency of <10% generally observed for each of these two subtypes (3).

### Genomic Comparison of C versus S Components

Next, we aimed to decipher the molecular mechanisms distinguishing the C and S components. To ensure the robustness of this analysis, we excluded mixed samples and selected only the five tumors for which paired samples were histologically classified as carcinoma or sarcoma (or undifferentiated): P01, P02, P05, P12, P15 (Fig. 1B). Comparison of genomic alterations identified in both samples of the same tumor revealed a substantial number of shared events. Indeed, genomic features displayed a high rate of common events between C/S-U components, with medians of 52% CNA (from 29% to 95%), 36% SV (from 0% to 61%), 59% SNV (from 26% to 83%), and 45% indel (from 29% to 66%; Supplementary Fig. S10).

Furthermore, mutational signature analysis corroborated that both samples from the same tumor globally shared identical mutational processes, further emphasizing the clonal evolution of CS stemming from a common origin of the two components.

### Transcriptomic and Epigenetic Analysis of C versus S Components

**FIGURE 3:**
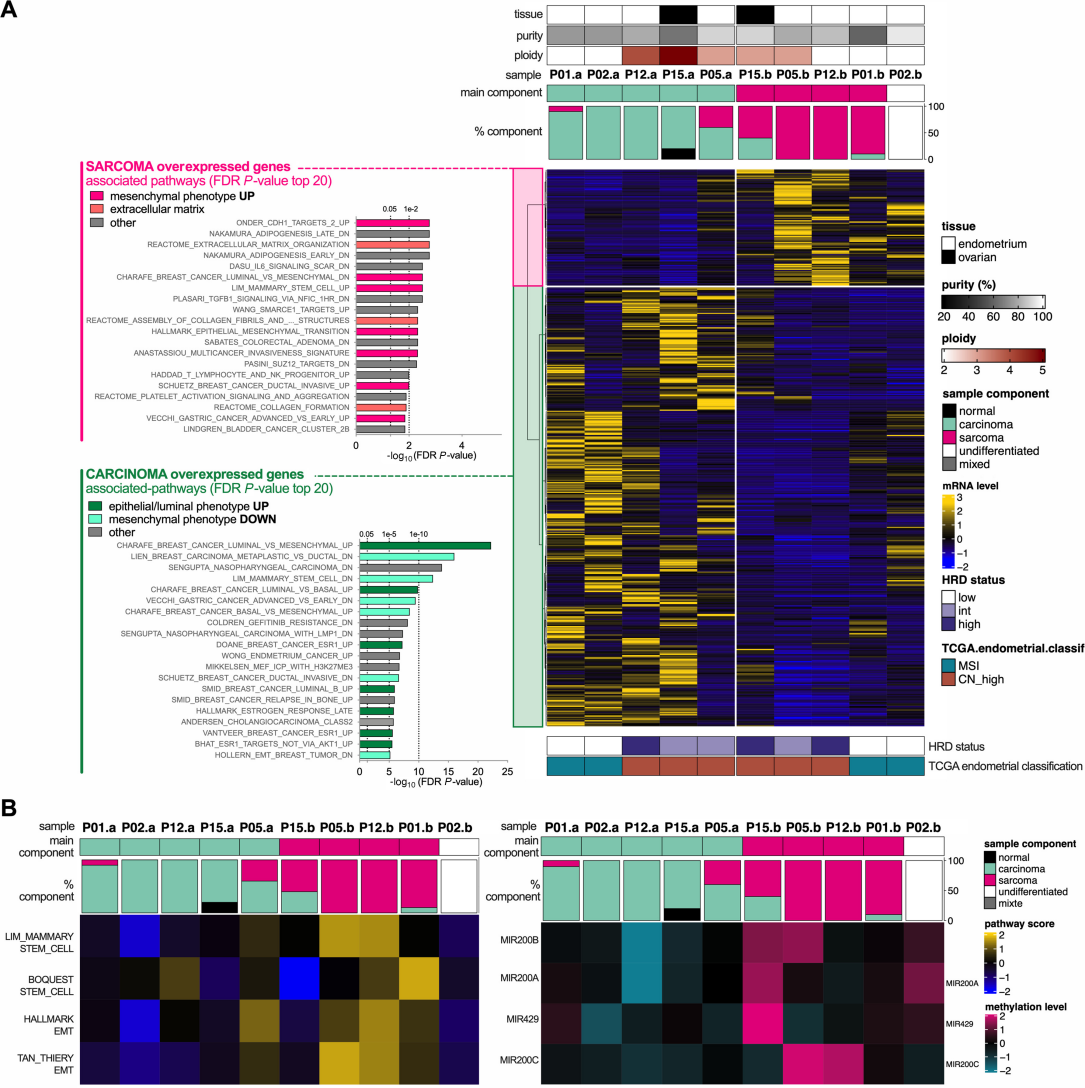
Differential expression and methylation analysis (C vs. S). **A,** Heatmap and pathway overrepresentation analysis of differentially expressed genes between C and S (or undifferentiated) components of the 10 samples derived from the five selected CS tumors. Gene clustering method: Ward's; distance: Spearman. FDR: false discovery rate. **B,** Heatmaps of EMT and stemness pathways scores (left) and methylation level of EMT-related miRNAs (right). mRNA level: scaled TPM. Pathway scores: scaled ssGSEA scores. Methylation level: scaled beta-values.

To identify nongenomic events underlying the phenotypic discrepancies observed between C and S samples, paired-differential transcriptomic and epigenetic analyses were conducted on the five selected tumors (P01, P02, P05, P12, P15). Differential expression analysis highlighted 363 overexpressed genes in C tissue (compared with S/U), associated to epithelial phenotype-related pathways (Fig. 3A; Supplementary Table S2). Inversely, 97 genes overexpressed in S/U tissue (compared with C) were linked to pathways related to the mesenchymal phenotype and extracellular matrix (ECM) remodeling (Fig. 3A; Supplementary Table S2). In accordance with these results, differential methylation analysis revealed an enrichment in ECM-related genes among those significantly hypomethylated in S/U samples (Supplementary Fig. S11; Supplementary Table S3). Concordant with these observations, S/U samples displayed elevated transcriptional scores for EMT (Hallmark EMT and Tan_Thiery EMT; refs. 22, 27) and stemness (Lim mammary stem cell and Boquest stem cell) -related pathways (refs. 24, 25; Fig. 3B). Finally, in S/U samples, a high level of methylation was observed for miRNAs such as the miR200 family, expression of which is typically associated with maintenance of the epithelial phenotype (Fig. 3B). Altogether, these results highlighted the major role of the EMT process in phenotypic divergence between C and S at the transcriptional level.

### Clonal History of CS Tumors

**FIGURE 4:**
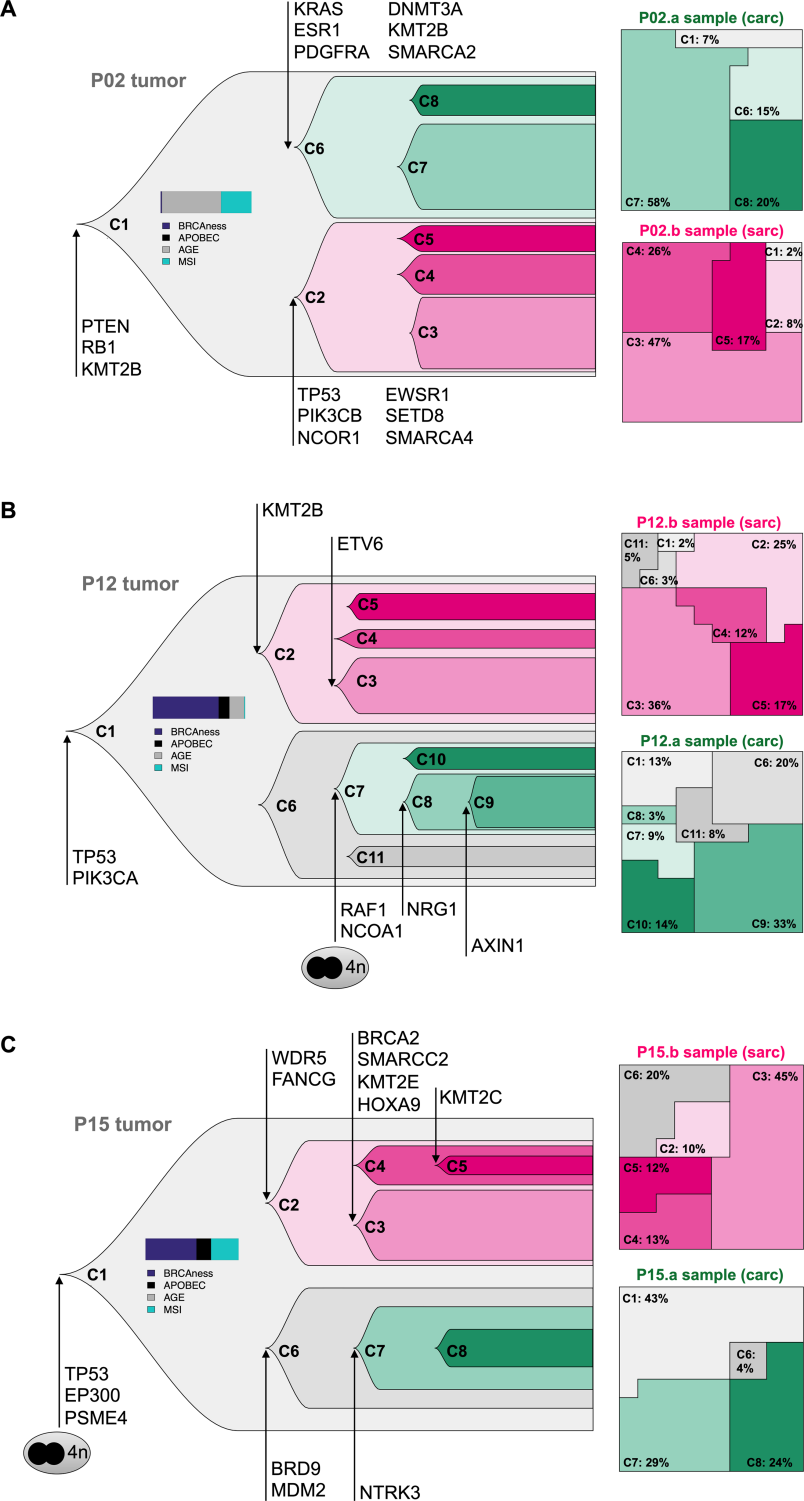
Evolutionary histories of CS. Clonal lineage histories of P02 MSI phenotype tumor (**A**), P12 BRCAness-phenotype tumor (**B**), and P15 ovarian bilateral tumor (**C**). Left, Clonal lineage inference for the whole tumor: subclonal populations (Ci), main cancer gene alterations, genome doubling events, and representative mutational signature proportions are represented. Right, Subclonal population frequencies projection in each tumor sample. Clones colored in gray are found in both derived samples, pink (or green) clones are specific to sarcoma- (or carcinoma-) derived samples, respectively.

Evolutionary histories of CS tumors have been reconstructed through the identification of subpopulations of cancer cells using the large number of somatic mutations and accurate estimation of copy-number changes provided by WGS data in our cohort (Fig. 4; Supplementary Fig. S12–S16). To build the phylogeny of subclonal populations, a two-step strategy combining both CNA and point mutations was used: step one consisted of the identification of CNA subclonal populations in each sample and step two relied on the incorporation of allelic frequencies of somatic mutations.

This analysis predicted that CS were composed of several subclones (from 7 to 10), indicating an intrinsic heterogeneity within these tumors (Fig. 4; Supplementary Fig. S12–S16). In each reconstructed tumor phylogeny, a clonal population (C1) was identified, harboring mutations present in all tumor cells, thus indicating a common origin of both components. VAFs from the C1 clonal population displayed similar distribution between both components, reflecting shared alterations rather than a cross-contamination bias (Fig. 4; Supplementary Fig. S12–S16).

To further understand individual evolutionary trajectories, mutation events occurring in each subclonal population were explored.

The MSI-related P02 tumor originated through alterations in cell cycle, PI3K, and CRG-related pathways (*RB1*, *PTEN*, and *KMT2B* mutations in C1 clonal population), while *TP53* mutation occurred specifically in the sarcomatous-related subclonal population C2 (Fig. 4A; Supplementary Fig. S12). Likewise, the other MSI-driven patient (P01) showed a similar tumor natural history (Supplementary Fig. S13).

P12 tumor, associated with the BRCAness-related mutational process, displayed clonal point mutations of *TP53* and *PIK3CA*. Interestingly, alterations in CRG, typically associated with features of cellular plasticity emerged not only in the S component but also in the C component (*KMT2B* and *NCOA1* mutations in C2 and C7 subpopulations, respectively; Fig. 4B; Supplementary Fig. S14). Similarly, P05 tumor, related to APOBEC mutational activity, was characterized by mutations in *TP53* and *PIK3CA* driver genes among the C1 clonal population and alterations in CRG in both components (Supplementary Fig. S15).

Finally, the P15 ovarian tumor, for which the C and S samples were collected from the left and right ovaries, respectively, arose from whole-genome doubling along with *TP53* clonal mutation and exhibited a BRCAness signature (HRD), concordant with an elevated genomic instability previously reported in serous high-grade ovarian cancers (Fig. 4C; Supplementary Fig. S16).

In line with P01, P02, P05, and P12 tumor histories, genomic alterations of CRG occurred in both C and S components of the P15 tumor, emphasizing the potential role of chromatin remodeling processes in CS tumorigenesis. Although common genomic features have been conjointly evidenced in CS tumors (*TP53*, *PI3K*), they were indiscriminately found in either C, S, or both components, suggesting that alterations in these pathways could not be hypothesized as a driver event of C-S phenotypic divergence. Similarly, constant proportions of mutational signatures have been identified in every distinct subclonal population within each tumor, illustrating a stable landscape of mutational processes all along tumor evolution (Supplementary Fig. S12–S16).

Altogether, our data illustrated that C and S samples from each tumor were concomitantly composed of both ancestral cell populations together with component-specific subclones, indicating a common population of origin for both components followed by distinct evolutionary trajectories.

## Discussion

In this study, we have provided for the first time a detailed characterization of the genomic landscape of CS using WGS. Our approach led us to identify a high frequency of shared somatic mutations (SV and indel) and additional complex genomic alterations (CNA and SV) between the C and S components. The extent of shared genetic events allowed us to emphasize previous findings supporting a monoclonal origin for CS tumors that disproves the collision theory.

Given the high frequency of genetic alterations observed, we first aimed to decipher whether CS heterogeneity is indeed driven by a genetic trigger. Paradoxically, while we could identify mutations belonging to key biological pathways that typically drive the hallmarks of tumorigenesis, none of these mutations seemed to discriminate systematically between the C and S components. More specifically, we identified five distinct molecular pathways that were altered in over 25% of our tumor cohort: TP53, PI3K/AKT, cell cycle, CRG, and RAS. These pathways represented genes that have central roles in regulating the cell cycle, cell growth, proliferation (e.g., *TP53*, *RB1*, *PTEN*, *PIK3CA*), chromatin remodeling (e.g., *SMARCA4*, *ARID1A*, *ARID1B*), chromatin compaction, and gene expression (e.g., *HDAC*, *KMT2B*). Aberration of genes like *TP53* or those involved in chromatin remodeling is known to disturb chromatin homeostasis and increase the permissiveness or plasticity of chromatin, which can in turn lower the energy barriers that are otherwise necessary to prevent changes in cellular state (34). However, in our group of tumor samples, we reported that frequent mutations in CRG or *TP53* were not limited to the S component but shared with the C component, supporting the hypothesis that the phenotypic switch of cellular state from C to S could be driven by an enhanced plasticity rather than by a specific and recurrent genetic alteration. This hypothesis was further supported by two arguments: (i) at the sample level, decomposition of mutational signatures computed from any tumor subpopulation was equivalent to the decomposition of the whole tumor, corroborating the fact that none of the mutational processes tend to drive the C to S phenotypic transition; and (ii) alterations in the five most frequently altered pathways were identified either in the common population or were acquired in a varied sequence order during the evolutionary process. We could therefore suggest, as hinted previously, that the CS plasticity switch might not be driven by a specific genetic trigger (3).

Upon investigating whether CS divergence could instead be driven by an epigenetic trigger, data from RNA-seq and DNA methylation analysis revealed substantial differences between the C and S components. Most notably, we found that small noncoding tumor suppressor miRNAs such as the miR-200 family are expressed in C cells (hypomethylated *miR200* promoter status), while being repressed in S cells (hypermethylated *miR200* promoter status) as reported previously (Fig. 3B; refs. 3, 32, 35). In accordance with the well-characterized role of *miR200* in EMT regulation, our differential expression analysis of C and S samples identified key mesenchymal genes and ECM remodeling genes to be overexpressed in S cells, while key genes of the epithelial phenotype including e-cadherin (*CDH1*) were upregulated in C cells (5). The collective genetic and epigenetic landscape of CS tumors characterized in our study reveals that there is no recurrent genetic event discriminating the C and S components, rather involves major differences in epithelial and mesenchymal transcriptomic signatures.

On the basis of our results, we hypothesize that while mutations affecting *TP53* and CRG impart an inherent plasticity and instability to epithelial cells making them “prone” to transdifferentiation early on in tumor evolution, the transdifferentiation process in itself is directly mediated by environmental cues. In this context, while all epithelial cells are potentially capable of evolving toward an S cell fate owing to their genetic heterogeneity, only those that encounter the necessary environmental cues would undergo the plasticity switch, both components would then continue to accumulate mutations, giving rise to C- and S-specific evolutionary trajectories. Our results place well the derivation of the S component from C cells in later stages of tumor evolution, possibly linked to the increasing complexity of the tumor microenvironment that exposes EMT-prone C cells to an abundance of EMT-inducing environmental signals.

The currently genomics-centric approaches to CS treatment mostly rely on mutational signatures identified by WGS/WES to define actionable gene targets, stratify patients by molecular aberration subtype and deliver individualized therapies. In light of the proposed impact of the tumor microenvironment on CS heterogeneity, the difficulty in treating aggressive tumors like CS can no longer be regarded as a sole consequence of drug resistance conferred through mutations in minor clonal populations. Instead, we must also acknowledge the role of microenvironmental stresses that can enhance cellular plasticity, allowing cells to acquire more resistant phenotypes.

## Supplementary Material

Supplementary Table 1 Curated list of chromatin remodeling genes.

Supplementary Table 2 List of differentially expressed genes (paired analysis of 5 selected tumors).

Supplementary Table 3 List of differentially methylated gene promoters (paired analysis of 5 selected tumors).

Figure S1 Collection and selection of tumor samples.

Figure S2 Heterogeneity of genomic rearrangements in carcinosarcoma.

Figure S3 Clustering of CS mutational signatures with COSMIC signatures.

Figure S4 APOBEC-related kataegis event.

Figure S5 BRCAness feature: tandem duplicator phenotype.

Figure S6 Alteration of genes of TP53, PI3K and cell cycle pathways in uterine and ovarian CS.

Figure S7 Alteration of chromatin remodeling genes in uterine and ovarian CS.

Figure S8 Alteration of genes of RAS pathway in uterine and ovarian CS.

Figure S9 Clustering of somatic copy number alterations profiles.

Figure S10 Analysis of common genomic events between paired tumor samples.

Figure S11 Differential methylation analysis (C vs S).

Figure S12 Complete clonal evolution of P02 tumor.

Figure S13 Complete clonal evolution of P01 tumor.

Figure S14 Complete clonal evolution of P05 tumor.

Figure S15 Complete clonal evolution of P12 tumor.

Figure S16 Complete clonal evolution of P15 tumor.
