# Supplementary material for: Dissecting the Origin of Heterogeneity in Uterine and Ovarian Carcinosarcomas
Source: Cancer Res Commun. 2023 May 10;3(5):830–41. doi: 10.1158/2767-9764.CRC-22-0520 (PMC10171113; doi:10.1158/2767-9764.CRC-22-0520)
Supplement: Figure S1 — Collection and selection of tumor samples. [file crc-22-0520-s04.pdf]

Figure S1

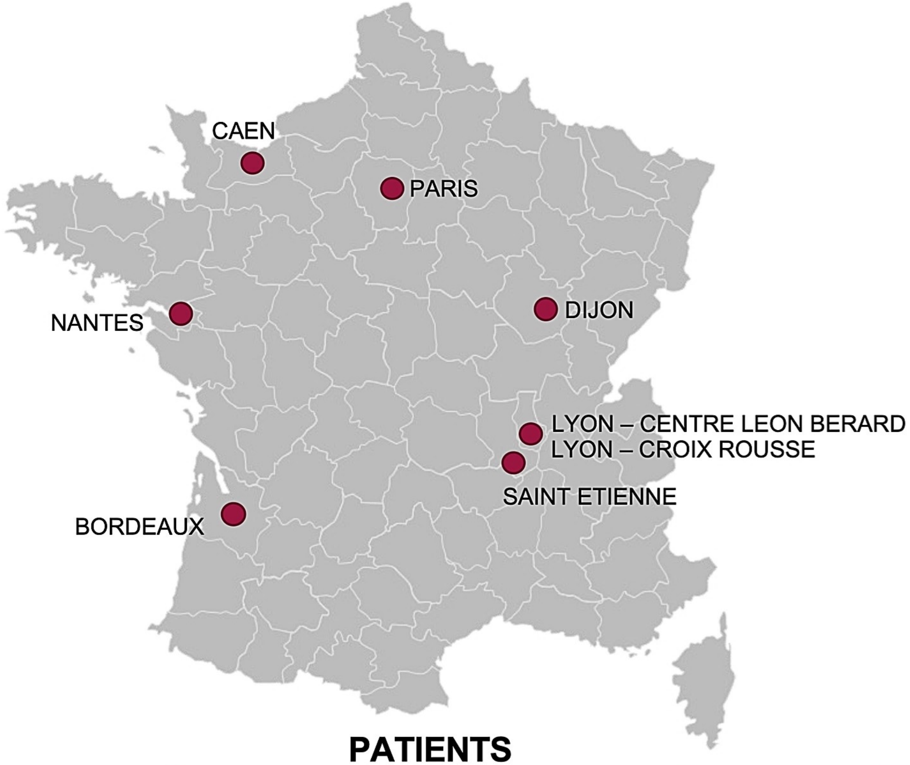

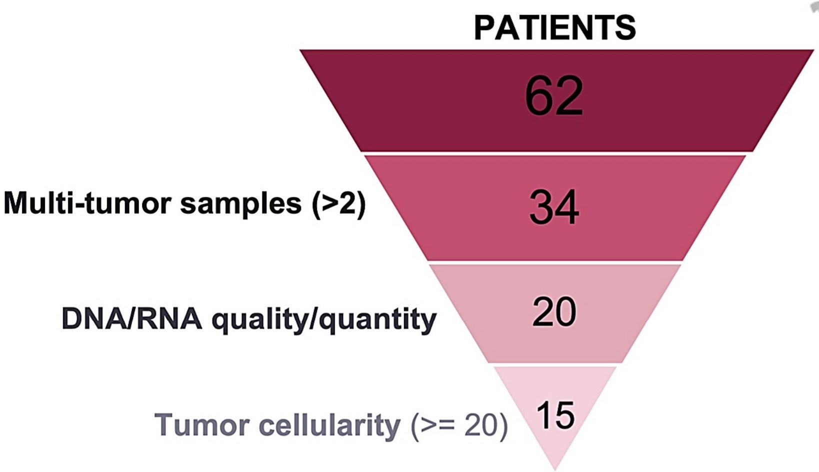

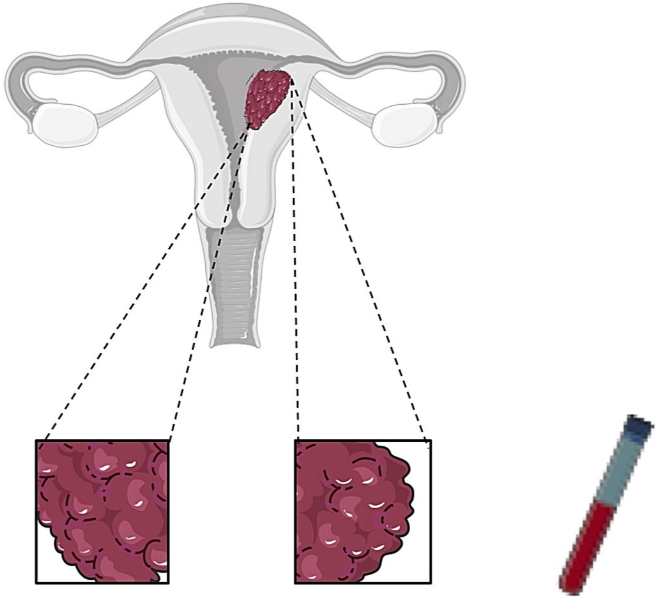

	Tumor region **a**	Tumor region **b**	Blood sample
Whole Genome Sequencing	x	x	x
Genotyping array	x	x	x
RNA Sequencing	x	x	
Methylation array	x	x	

Supplementary Figure 1. Collection and selection of tumor samples. (Left) Tumor tissue and matched blood samples recruitment and selection criteria. (Right) Description of the molecular analysis conducted on all sample types.
