# Supplementary material for: Dissecting the Origin of Heterogeneity in Uterine and Ovarian Carcinosarcomas
Source: Cancer Res Commun. 2023 May 10;3(5):830–41. doi: 10.1158/2767-9764.CRC-22-0520 (PMC10171113; doi:10.1158/2767-9764.CRC-22-0520)
Supplement: Figure S2 — Heterogeneity of genomic rearrangements in carcinosarcoma. [file crc-22-0520-s05.pdf]

Figure S2

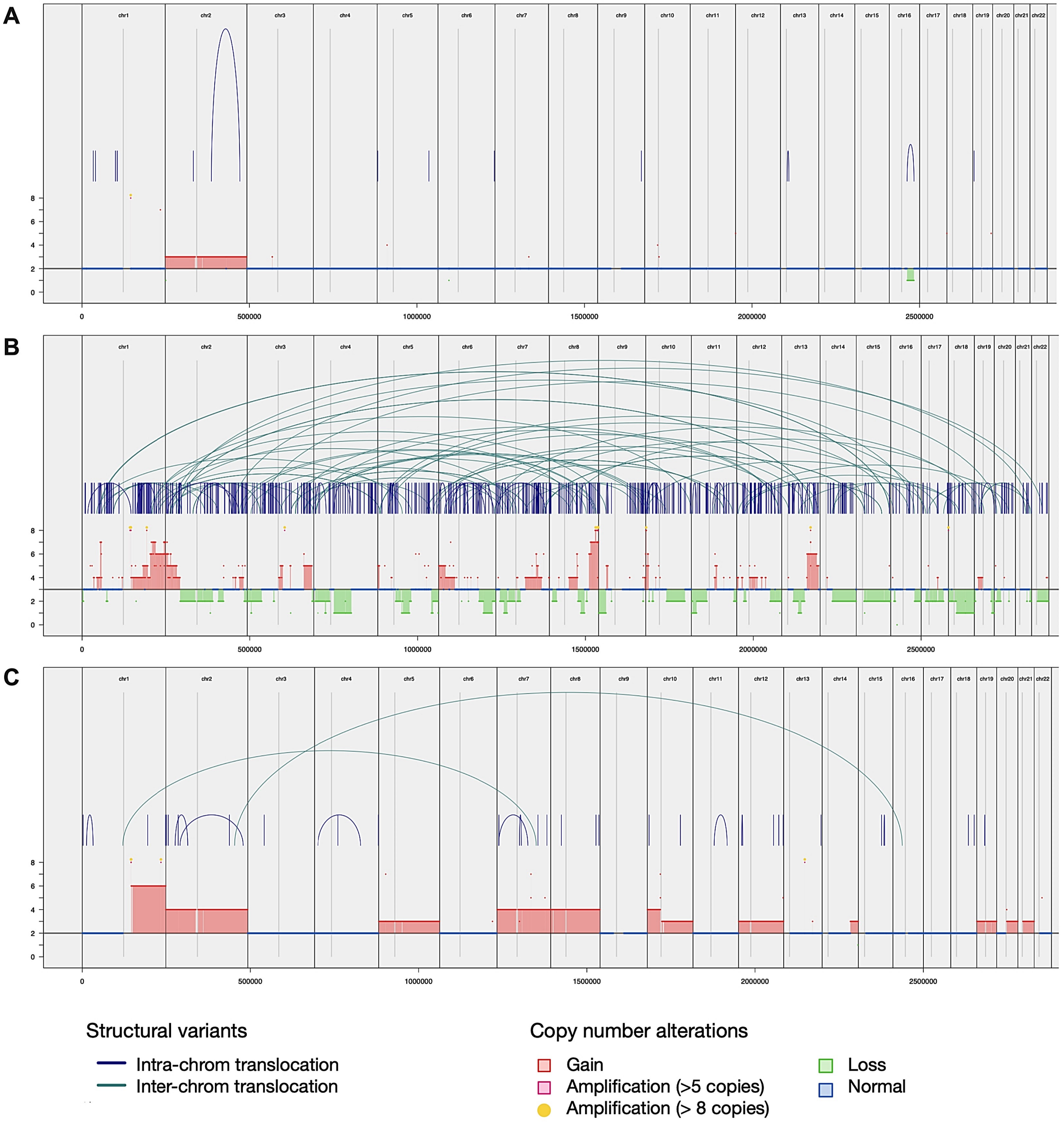
A
B
C
chr1 chr2 chr3 chr4 chr5 chr6 chr7 chr8 chr9 chr10 chr11 chr12 chr13 chr14 chr15 chr16 chr17 chr18 chr19 chr20 chr21 chr22
0
2
4
6
8
0
500000
1000000
1500000
2000000
2500000
Structural variants
Intra-chrom translocation
Inter-chrom translocation
Copy number alterations
Gain
Amplification (>5 copies)
Amplification (> 8 copies)
Loss
Normal

Supplementary Figure 2. Heterogeneity of genomic rearrangements in carcinosarcoma. Pangenomic representation of CNA (losses, gains and amplifications) and SV (intra and inter-chromosomal translocations) of samples associated with various histological subtypes: **A**, P01.a: endometrium, endometrioid, MSI phenotype, *TP53* wild-type. **B**, P13.a: ovarian, serous, HRD-high phenotype (TDP positive), *TP53* mutated. **C**, P10.a: endometrium, endometrioid, *TP53* wild-type.
