# Supplementary material for: Dissecting the Origin of Heterogeneity in Uterine and Ovarian Carcinosarcomas
Source: Cancer Res Commun. 2023 May 10;3(5):830–41. doi: 10.1158/2767-9764.CRC-22-0520 (PMC10171113; doi:10.1158/2767-9764.CRC-22-0520)
Supplement: Figure S3 — Clustering of CS mutational signatures with COSMIC signatures. [file crc-22-0520-s06.pdf]

Figure S3

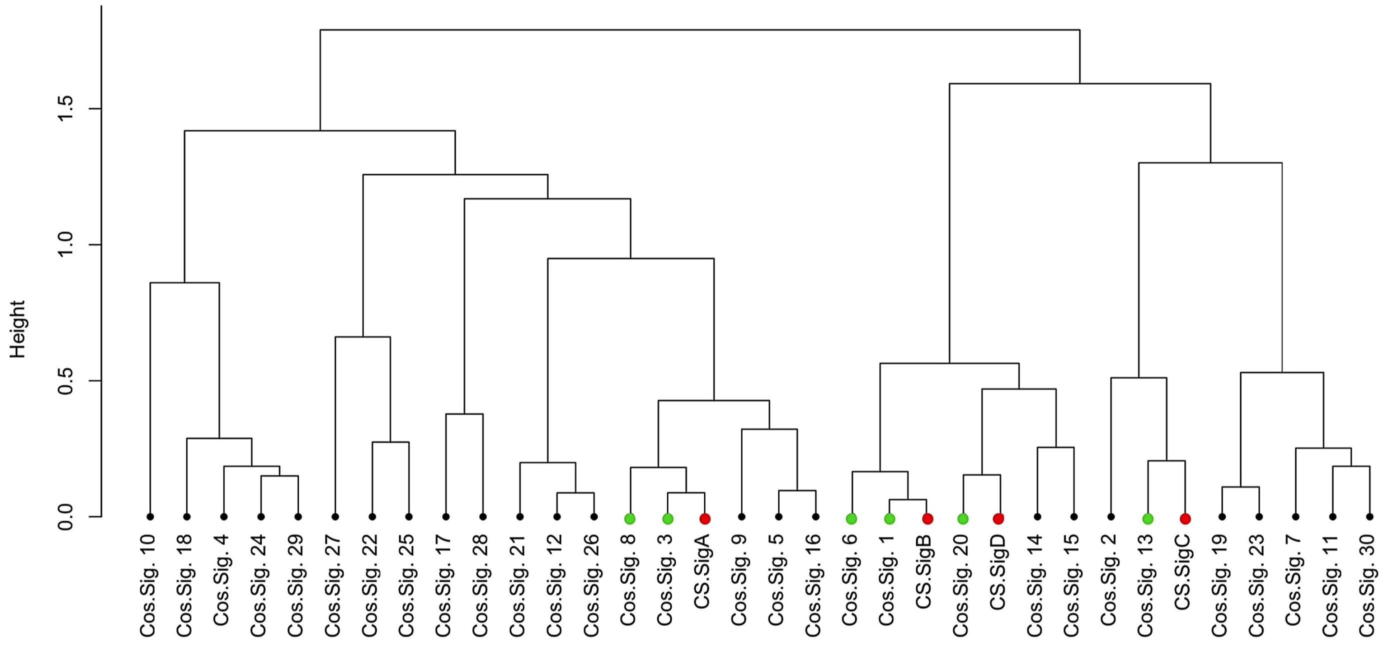

CS de-novo mutational signatures

Closest COSMIC signatures

CS.SigA : BRCAness-related
CS.SigB : Age-related
CS.SigC : APOBEC-related
CS.SigD : MSI-related

Supplementary Figure 3. Clustering of CS mutational signatures with COSMIC signatures.

Clustering tree of the four mutational signatures deciphered in CS samples (red dots) with the 30 COSMIC signatures. Aetiologies of the four CS mutational signatures are inferred from the closest COSMIC signature in the clustering (green dots). Clustering method: Ward's; distance: 1-cosine similarity.
