# Supplementary material for: Dissecting the Origin of Heterogeneity in Uterine and Ovarian Carcinosarcomas
Source: Cancer Res Commun. 2023 May 10;3(5):830–41. doi: 10.1158/2767-9764.CRC-22-0520 (PMC10171113; doi:10.1158/2767-9764.CRC-22-0520)
Supplement: Figure S4 — APOBEC-related kataegis event. [file crc-22-0520-s07.pdf]

Figure S4

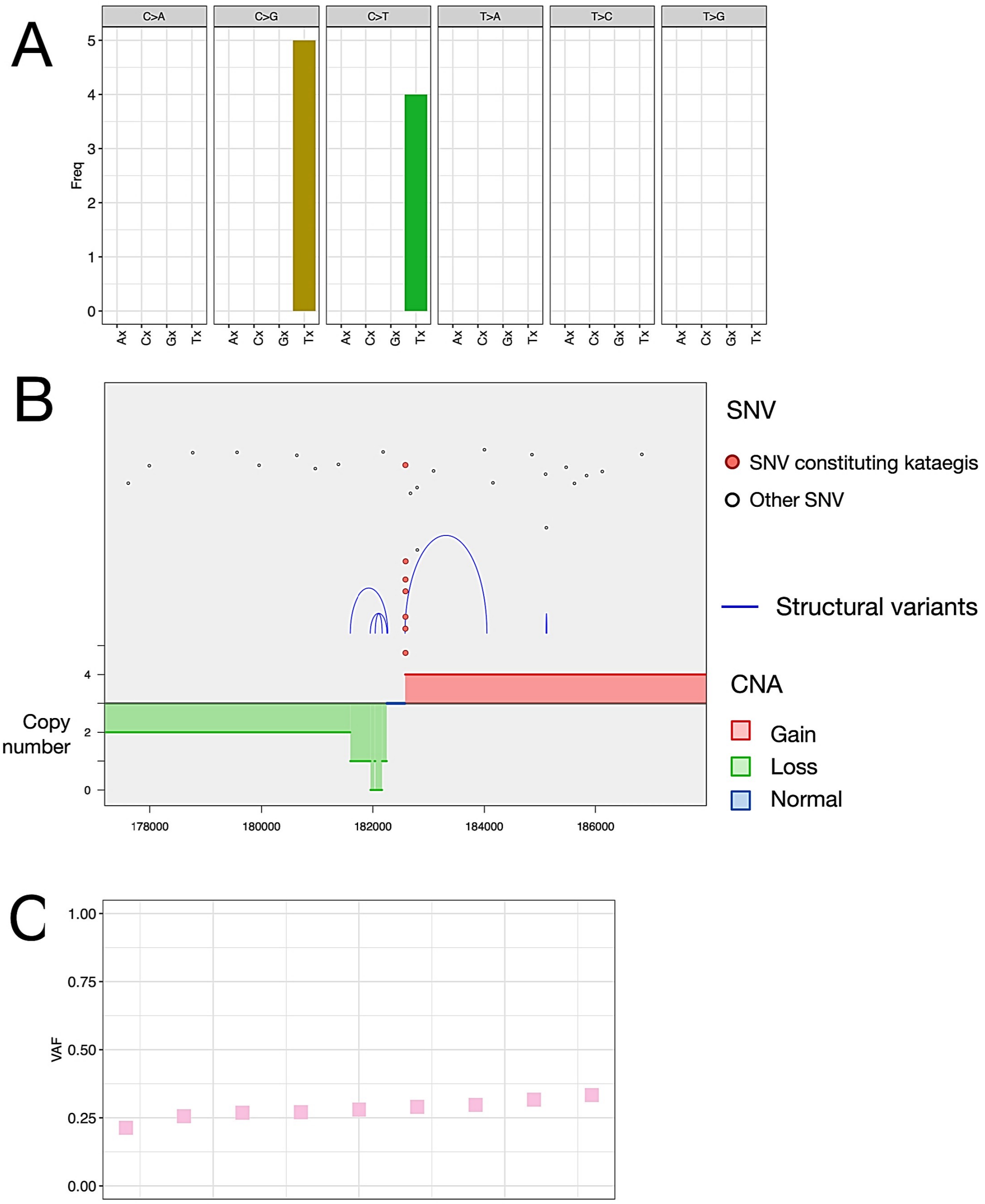
A
C>A
C>G
C>T
T>A
T>C
T>G
Freq
Ax Cx Gx Tx
B
SNV
SNV constituting kataegis
Other SNV
Structural variants
CNA
Gain
Loss
Normal
Copy number
178000
180000
182000
184000
186000
C
VAF
1.00
0.75
0.50
0.25
0.00

Supplementary Figure 4. APOBEC-related kataegis event. APOBEC-related kataegis event in sample P05.a. **A**, Mutational spectrum of the eight SNV constituting to the kataegis event is enriched in TC>G and TC>T substitutions, characteristic of APOBEC-mediated mutagenesis. **B**, Kataegis events are retained if located near a genomic rearrangement. **C**, Homogeneous variant allele frequencies (VAF) of these eight SNV suggest that they occurred during the same genetic event.
