# Supplementary material for: Dissecting the Origin of Heterogeneity in Uterine and Ovarian Carcinosarcomas
Source: Cancer Res Commun. 2023 May 10;3(5):830–41. doi: 10.1158/2767-9764.CRC-22-0520 (PMC10171113; doi:10.1158/2767-9764.CRC-22-0520)
Supplement: Figure S5 — BRCAness feature: tandem duplicator phenotype. [file crc-22-0520-s08.pdf]

Figure S5

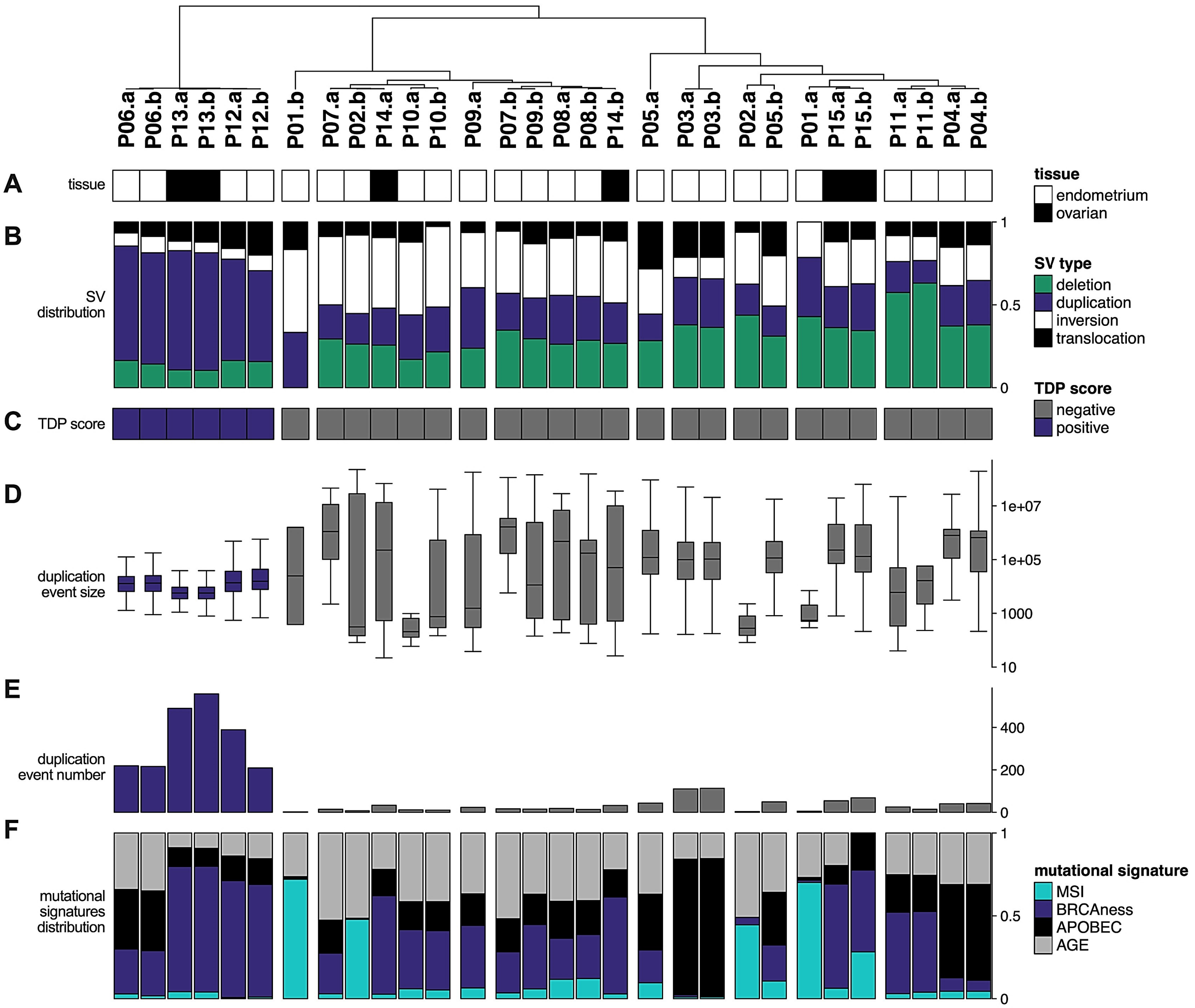

P06.a
P06.b
P13.a
P13.b
P12.a
P12.b
P01.b
P07.a
P02.b
P14.a
P10.a
P10.b
P09.a
P07.b
P09.b
P08.a
P08.b
P14.b
P05.a
P03.a
P03.b
P02.a
P05.b
P01.a
P15.a
P15.b
P11.a
P11.b
P04.a
P04.b
A
tissue
B
SV distribution
C
TDP score
D
duplication event size
E
duplication event number
F
mutational signatures distribution
1
0.5
0
1e+07
1e+05
1000
10
400
200
tissue
endometrium
ovarian
SV type
deletion
duplication
inversion
translocation
TDP score
negative
positive
mutational signature
MSI
BRCAness
APOBEC
AGE

Supplementary Figure 5. BRCAness feature: tandem duplicator phenotype. Clustering of SV type proportions. Clustering method: Ward's; distance: Pearson. **A**, Sample tissue origin. **B**, Distribution of SV types. **C**, TDP score. **D**, Distribution of duplication events sizes (bp) *per* sample (log-scaled). **E**, Number of duplication events *per* sample. **F**, Proportion of each of four mutational signatures deciphered in the whole cohort.
