# Supplementary material for: Dissecting the Origin of Heterogeneity in Uterine and Ovarian Carcinosarcomas
Source: Cancer Res Commun. 2023 May 10;3(5):830–41. doi: 10.1158/2767-9764.CRC-22-0520 (PMC10171113; doi:10.1158/2767-9764.CRC-22-0520)
Supplement: Figure S6 — Alteration of genes of TP53, PI3K and cell cycle pathways in uterine and ovarian CS. [file crc-22-0520-s09.pdf]

Figure S6

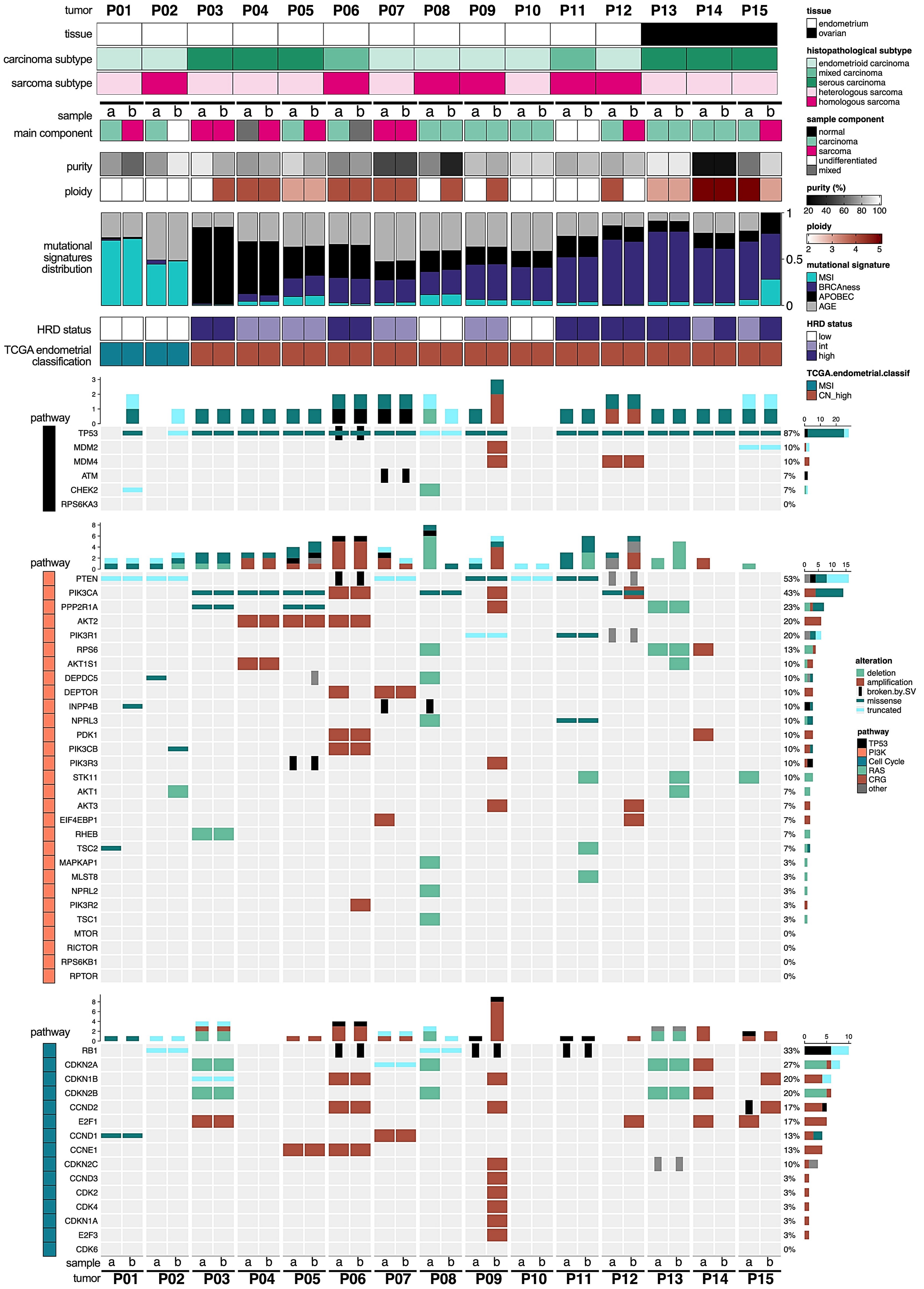

tumor
P01 P02 P03 P04 P05 P06 P07 P08 P09 P10 P11 P12 P13 P14 P15
tissue
carcinoma subtype
sarcoma subtype
sample
a b
main component
purity
ploidy
mutational signatures distribution
HRD status
TCGA endometrial classification
pathway
TP53 MDM2 MDM4 ATM CHEK2 RPS6KA3
PTEN PIK3CA PPP2R1A AKT2 PIK3R1 RPS6 AKT1S1 DEPDC5 DEPTOR INPP4B NPRL3 PDK1 PIK3CB PIK3R3 STK11 AKT1 AKT3 EIF4EBP1 RHEB TSC2 MAPKAP1 MLST8 NPRL2 PIK3R2 TSC1 MTOR RICTOR RPS6KB1 RPTOR
RB1 CDKN2A CDKN1B CDKN2B CCND2 E2F1 CCND1 CCNE1 CDKN2C CCND3 CDK2 CDK4 CDKN1A E2F3 CDK6
87% 10% 10% 7% 7% 0%
53% 43% 23% 20% 20% 13% 10% 10% 10% 10% 10% 10% 10% 10% 10% 7% 7% 7% 7% 7% 3% 3% 3% 3% 3% 0% 0% 0% 0%
33% 27% 20% 20% 17% 17% 13% 13% 10% 3% 3% 3% 3% 3% 0%
tissue
endometrium
ovarian
histopathological subtype
endometrioid carcinoma
mixed carcinoma
serous carcinoma
heterologous sarcoma
homologous sarcoma
sample component
normal
carcinoma
sarcoma
undifferentiated
mixed
purity (%)
20 40 60 80 100
ploidy
2 3 4 5
mutational signature
MSI
BRCAness
APOBEC
AGE
HRD status
low
int
high
TCGA.endometrial.classif
MSI
CN_high
alteration
deletion
amplification
broken.by.SV
missense
truncated
pathway
TP53
PI3K
Cell Cycle
RAS
CRG
other

Supplementary Figure 6. Alteration of genes of TP53, PI3K and cell cycle pathways in uterine and ovarian CS. Oncoprint of alterations identified in TP53, PI3K and cell cycle pathways from TCGA cancer pathways lists. The type of genomic alteration (deletion, amplification, fusion, broken by SV, missense, truncated) is described in the legend.
