# Supplementary material for: Dissecting the Origin of Heterogeneity in Uterine and Ovarian Carcinosarcomas
Source: Cancer Res Commun. 2023 May 10;3(5):830–41. doi: 10.1158/2767-9764.CRC-22-0520 (PMC10171113; doi:10.1158/2767-9764.CRC-22-0520)
Supplement: Figure S7 — Alteration of chromatin remodeling genes in uterine and ovarian CS. [file crc-22-0520-s10.pdf]

Figure S7

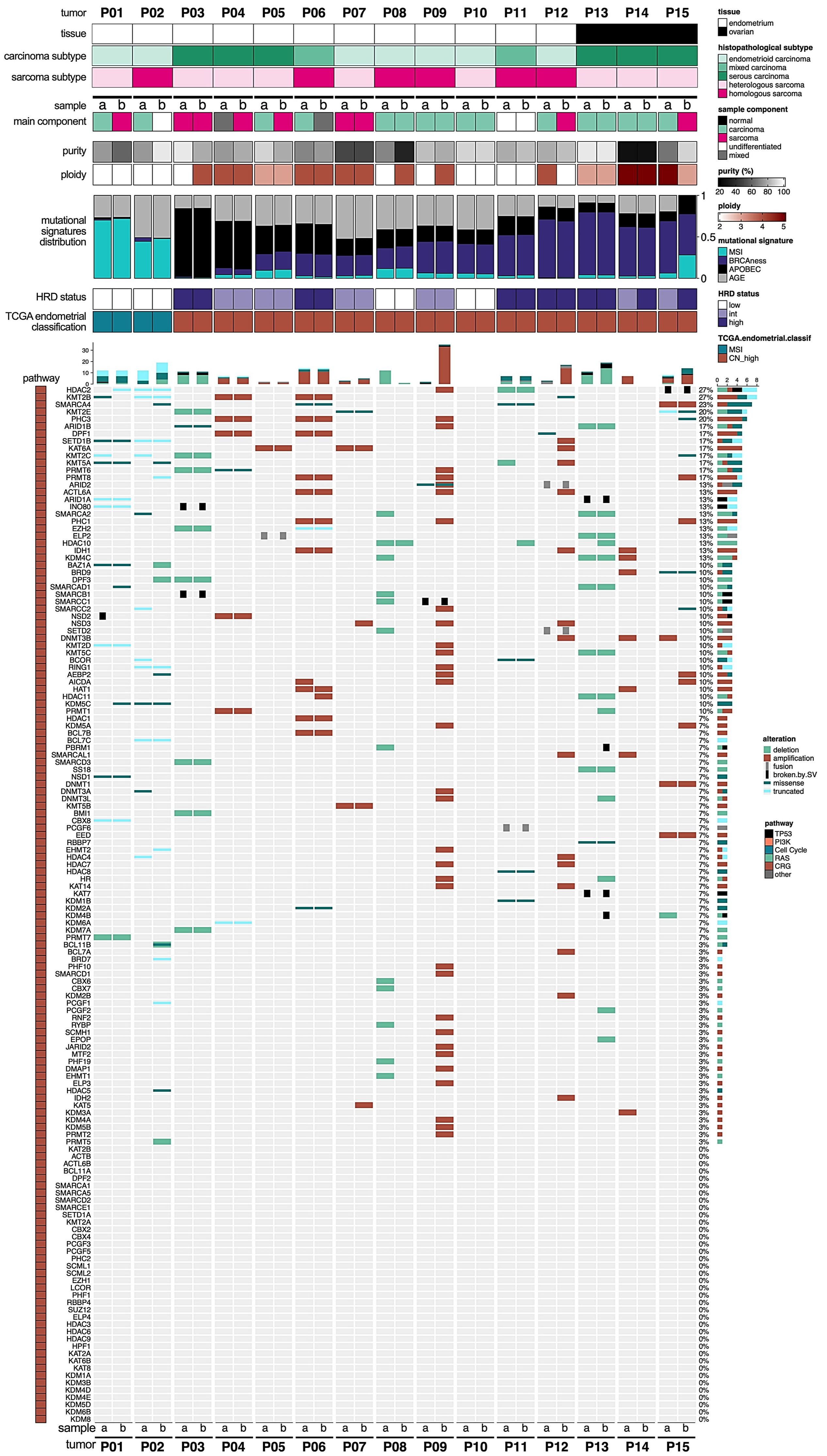
tumor
P01 P02 P03 P04 P05 P06 P07 P08 P09 P10 P11 P12 P13 P14 P15
tissue
carcinoma subtype
sarcoma subtype
sample
a b
main component
purity
ploidy
mutational signatures distribution
1
0.5
0
HRD status
TCGA endometrial classification
30
20
10
0
pathway
HDAC2
KMT2B
SMARCA4
KMT2E
PHC3
ARID1B
DPF1
SETD1B
KAT6A
KMT2C
KMT5A
PRMT6
PRMT8
ARID2
ACTL6A
ARID1A
INO80
SMARCA2
PHC1
EZH2
ELP2
HDAC10
IDH1
KDM4C
BAZ1A
BRD9
DPF3
SMARCAD1
SMARCB1
SMARCC1
SMARCC2
NSD2
NSD3
SETD2
DNMT3B
KMT2D
KMT5C
BCOR
RING1
AEBP2
AICDA
HAT1
HDAC11
KDM5C
PRMT1
HDAC1
KDM5A
BCL7B
BCL7C
PBRM1
SMARCAL1
SMARCD3
SS18
NSD1
DNMT1
DNMT3A
DNMT3L
KMT5B
BMI1
CBX8
PCGF6
EED
RBBP7
EHMT2
HDAC4
HDAC7
HDAC8
HR
KAT14
KAT7
KDM1B
KDM2A
KDM4B
KDM6A
KDM7A
PRMT7
BCL11B
BCL7A
BRD7
PHF10
SMARCD1
CBX6
CBX7
KDM2B
PCGF1
PCGF2
RNF2
RYBP
SCMH1
EPOP
JARID2
MTF2
PHF19
DMAP1
EHMT1
ELP3
HDAC5
IDH2
KAT5
KDM3A
KDM4A
KDM5B
PRMT2
PRMT5
KAT2B
ACTB
ACTL6B
BCL11A
DPF2
SMARCA1
SMARCA5
SMARCD2
SMARCE1
SETD1A
KMT2A
CBX2
CBX4
PCGF3
PCGF5
PHC2
SCML1
SCML2
EZH1
LCOR
PHF1
RBBP4
SUZ12
ELP4
HDAC3
HDAC6
HDAC9
HPF1
KAT2A
KAT6B
KAT8
KDM1A
KDM3B
KDM4D
KDM4E
KDM5D
KDM6B
KDM8
0 2 4 6 8
tissue
endometrium
ovarian
histopathological subtype
endometrioid carcinoma
mixed carcinoma
serous carcinoma
heterologous sarcoma
homologous sarcoma
sample component
normal
carcinoma
sarcoma
undifferentiated
mixed
purity (%)
20 40 60 80 100
ploidy
2 3 4 5
mutational signature
MSI
BRCAness
APOBEC
AGE
HRD status
low
int
high
TCGA.endometrial.classif
MSI
CN_high
alteration
deletion
amplification
fusion
broken.by.SV
missense
truncated
pathway
TP53
PI3K
Cell Cycle
RAS
CRG
other

Supplementary Figure 7. Alteration of chromatin remodeling genes in uterine and ovarian CS. Oncoprint of alterations identified in CRG (chromatin remodeling genes, custom genes list). The type of genomic alteration (deletion, amplification, fusion, broken by SV, missense, truncated) is described in the legend.
