# Supplementary material for: Dissecting the Origin of Heterogeneity in Uterine and Ovarian Carcinosarcomas
Source: Cancer Res Commun. 2023 May 10;3(5):830–41. doi: 10.1158/2767-9764.CRC-22-0520 (PMC10171113; doi:10.1158/2767-9764.CRC-22-0520)
Supplement: Figure S8 — Alteration of genes of RAS pathway in uterine and ovarian CS. [file crc-22-0520-s11.pdf]

Figure S8

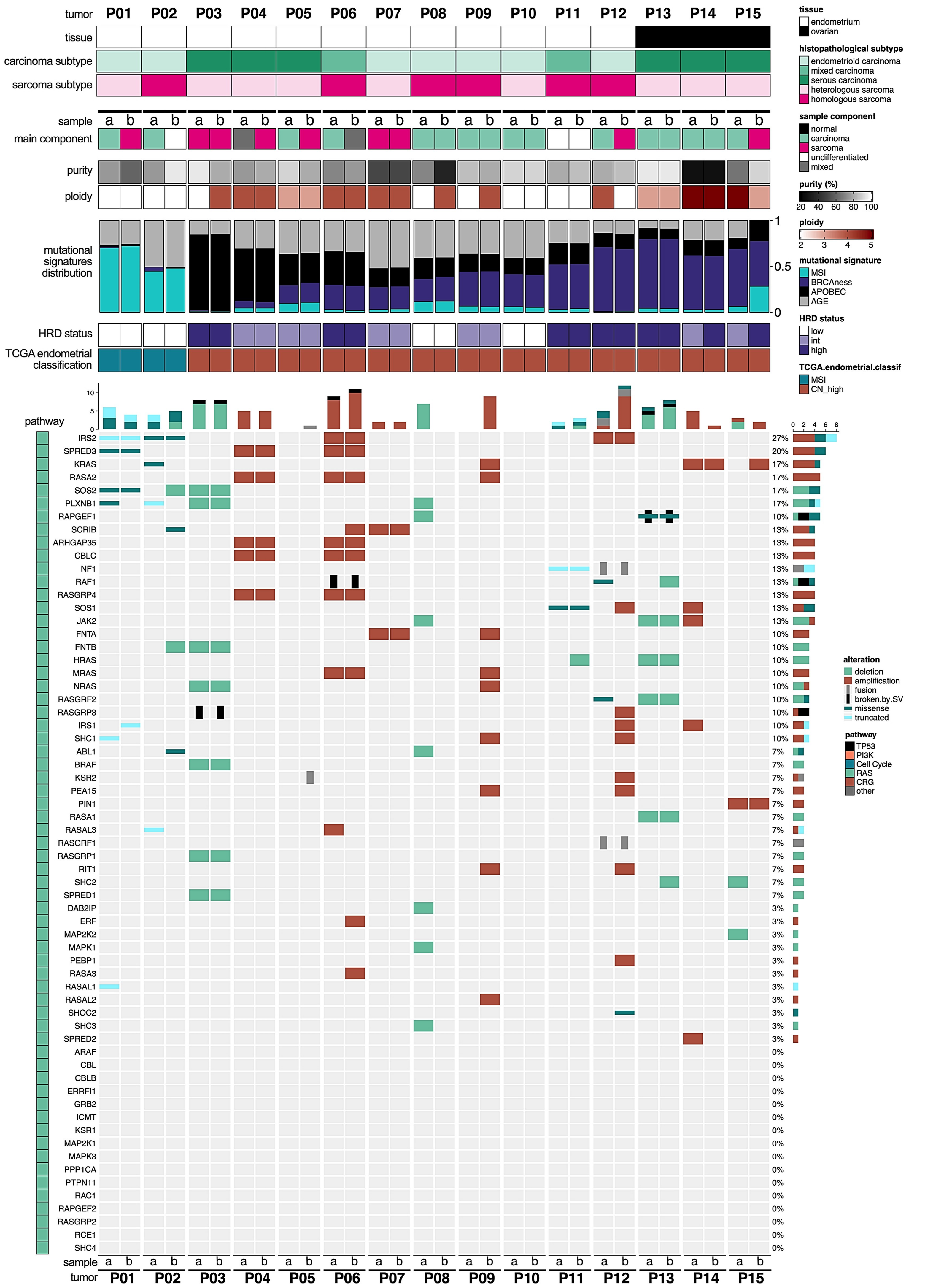

tumor
P01 P02 P03 P04 P05 P06 P07 P08 P09 P10 P11 P12 P13 P14 P15
tissue
carcinoma subtype
sarcoma subtype
sample
main component
purity
ploidy
mutational signatures distribution
HRD status
TCGA endometrial classification
pathway
IRS2
SPRED3
KRAS
RASA2
SOS2
PLXNB1
RAPGEF1
SCRIB
ARHGAP35
CBLC
NF1
RAF1
RASGRP4
SOS1
JAK2
FNTA
FNTB
HRAS
MRAS
NRAS
RASGRF2
RASGRP3
IRS1
SHC1
ABL1
BRAF
KSR2
PEA15
PIN1
RASA1
RASAL3
RASGRF1
RASGRP1
RIT1
SHC2
SPRED1
DAB2IP
ERF
MAP2K2
MAPK1
PEBP1
RASA3
RASAL1
RASAL2
SHOC2
SHC3
SPRED2
ARAF
CBL
CBLB
ERRFI1
GRB2
ICMT
KSR1
MAP2K1
MAPK3
PPP1CA
PTPN11
RAC1
RAPGEF2
RASGRP2
RCE1
SHC4
27%
20%
17%
17%
17%
17%
10%
13%
13%
13%
13%
13%
13%
13%
13%
10%
10%
10%
10%
10%
10%
10%
10%
10%
7%
7%
7%
7%
7%
7%
7%
7%
7%
7%
7%
7%
3%
3%
3%
3%
3%
3%
3%
3%
3%
3%
3%
0%
0%
0%
0%
0%
0%
0%
0%
0%
0%
0%
0%
0%
0%
0%
0%
tissue
endometrium
ovarian
histopathological subtype
endometrioid carcinoma
mixed carcinoma
serous carcinoma
heterologous sarcoma
homologous sarcoma
sample component
normal
carcinoma
sarcoma
undifferentiated
mixed
purity (%)
20 40 60 80 100
ploidy
2 3 4 5
mutational signature
MSI
BRCAness
APOBEC
AGE
HRD status
low
int
high
TCGA.endometrial.classif
MSI
CN_high
alteration
deletion
amplification
fusion
broken.by.SV
missense
truncated
pathway
TP53
PI3K
Cell Cycle
RAS
CRG
other

Supplementary Figure 8. Alteration of genes of RAS pathway in uterine and ovarian CS. Oncoprint of alterations identified in RAS pathway from TCGA cancer pathways lists. The type of genomic alteration (deletion, amplification, fusion, broken by SV, missense, truncated) is described in the legend.
