# Supplementary material for: Dissecting the Origin of Heterogeneity in Uterine and Ovarian Carcinosarcomas
Source: Cancer Res Commun. 2023 May 10;3(5):830–41. doi: 10.1158/2767-9764.CRC-22-0520 (PMC10171113; doi:10.1158/2767-9764.CRC-22-0520)
Supplement: Figure S9 — Clustering of somatic copy number alterations profiles. [file crc-22-0520-s12.pdf]

Figure S9

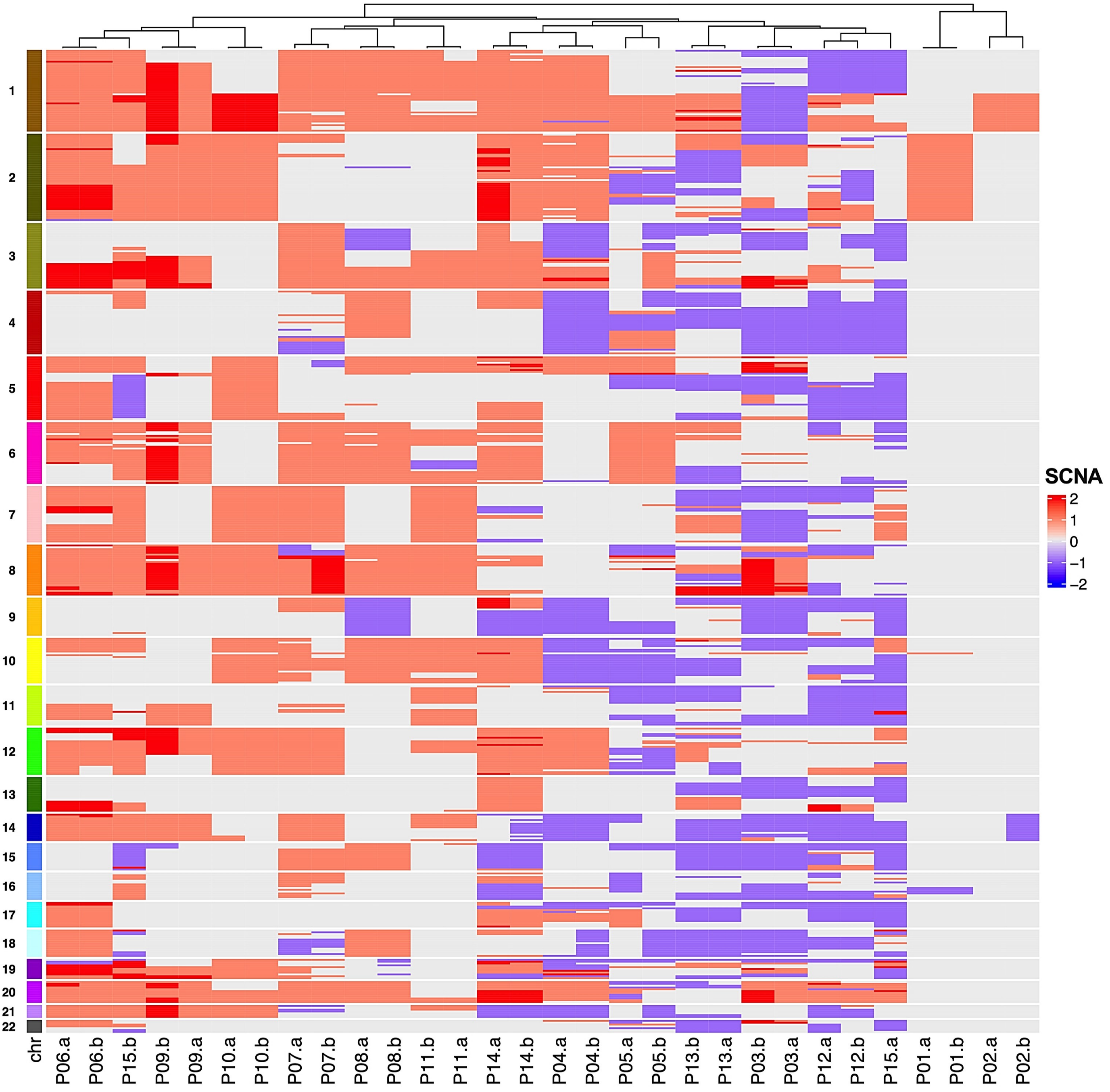

SCNA
2
1
0
−1
−2
chr
1
2
3
4
5
6
7
8
9
10
11
12
13
14
15
16
17
18
19
20
21
22
P06.a
P06.b
P15.b
P09.b
P09.a
P10.a
P10.b
P07.a
P07.b
P08.a
P08.b
P11.b
P11.a
P14.a
P14.b
P04.a
P04.b
P05.a
P05.b
P13.b
P13.a
P03.b
P03.a
P12.a
P12.b
P15.a
P01.a
P01.b
P02.a
P02.b

Supplementary Figure 9. Clustering of somatic copy number alterations profiles. Heatmap of somatic copy number alterations for each sample (columns) by chromosomal location (rows). Genome copy number alterations (CNA) were recoded as -2: homozygous loss, -1: loss, 0: neutral, 1: gain, 2: amplification, on windows of 5 Mb along the genomes; segments without any CNA across the samples were removed. Clustering method: Ward's; distance: binary.
