# Supplementary material for: Dissecting the Origin of Heterogeneity in Uterine and Ovarian Carcinosarcomas
Source: Cancer Res Commun. 2023 May 10;3(5):830–41. doi: 10.1158/2767-9764.CRC-22-0520 (PMC10171113; doi:10.1158/2767-9764.CRC-22-0520)
Supplement: Figure S10 — Analysis of common genomic events between paired tumor samples. [file crc-22-0520-s13.pdf]

Figure S10

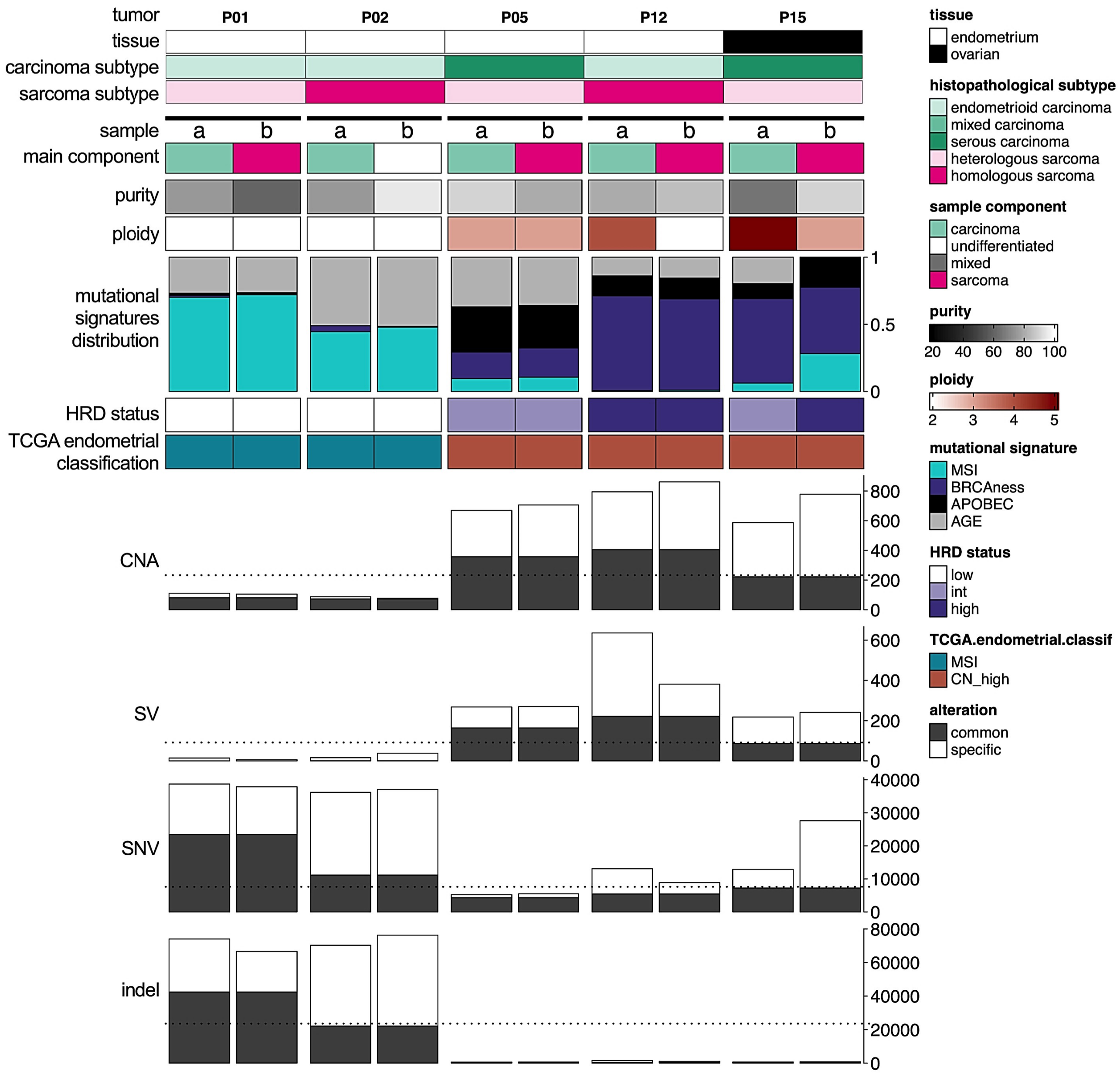

tumor
tissue
carcinoma subtype
sarcoma subtype
sample
main component
purity
ploidy
mutational signatures distribution
HRD status
TCGA endometrial classification
CNA
SV
SNV
indel
P01
P02
P05
P12
P15
a
b
1
0.5
0
800
600
400
200
0
600
400
200
0
40000
30000
20000
10000
0
80000
60000
40000
20000
0
tissue
endometrium
ovarian
histopathological subtype
endometrioid carcinoma
mixed carcinoma
serous carcinoma
heterologous sarcoma
homologous sarcoma
sample component
carcinoma
undifferentiated
mixed
sarcoma
purity
20 40 60 80 100
ploidy
2 3 4 5
mutational signature
MSI
BRCAness
APOBEC
AGE
HRD status
low
int
high
TCGA.endometrial.classif
MSI
CN_high
alteration
common
specific

Supplementary Figure 10. Analysis of common genomic events between paired tumor samples. Number of common and specific CNA (common: breakpoints within 2 Kb), SV (common: identical type and breakpoints), SNV and small indel between both samples of the five selected tumors.
