# Supplementary material for: Dissecting the Origin of Heterogeneity in Uterine and Ovarian Carcinosarcomas
Source: Cancer Res Commun. 2023 May 10;3(5):830–41. doi: 10.1158/2767-9764.CRC-22-0520 (PMC10171113; doi:10.1158/2767-9764.CRC-22-0520)
Supplement: Figure S11 — Differential methylation analysis (C vs S). [file crc-22-0520-s14.pdf]

Figure S11

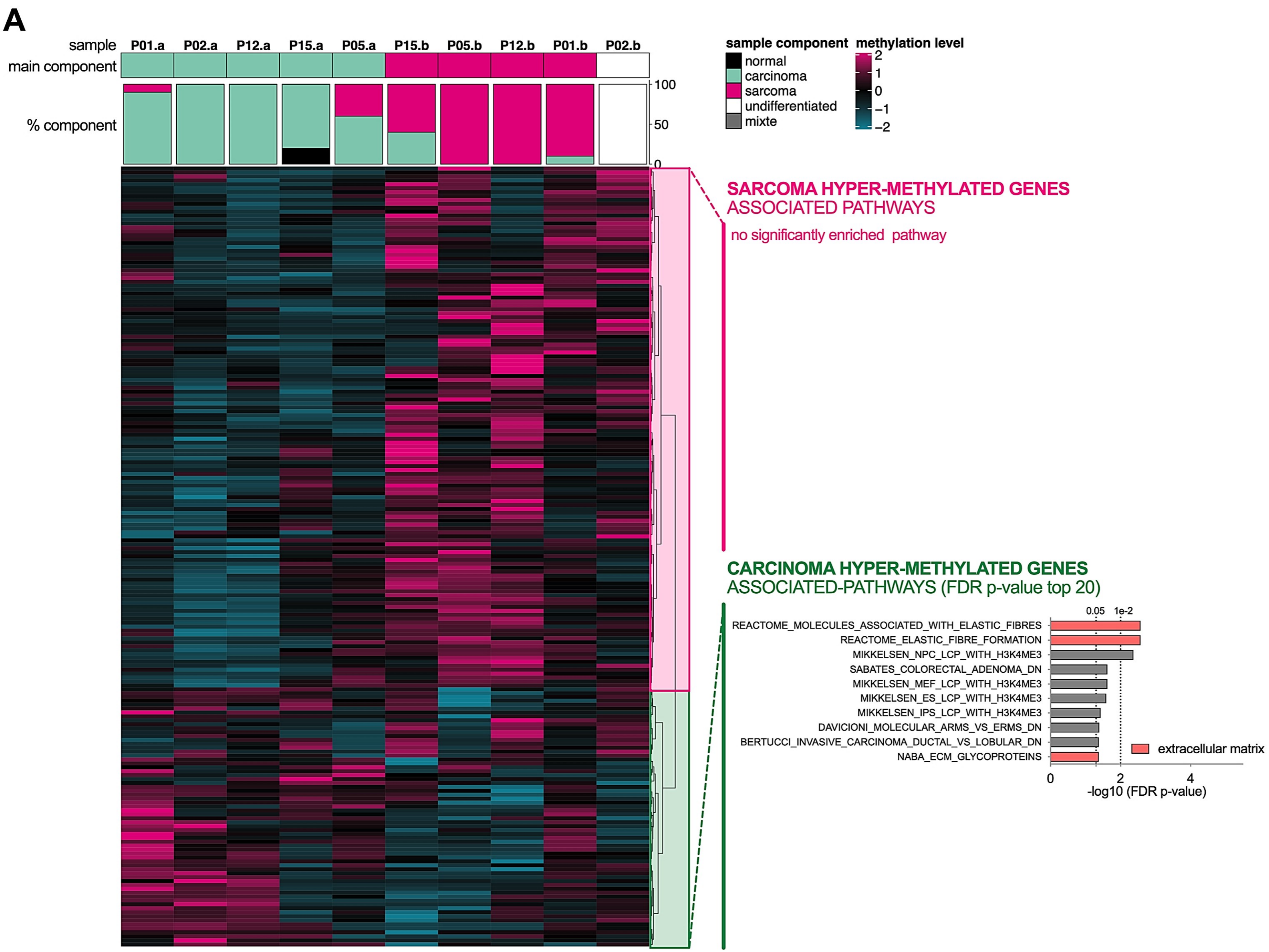

A
sample
P01.a
P02.a
P12.a
P15.a
P05.a
P15.b
P05.b
P12.b
P01.b
P02.b
main component
% component
100
50
0
sample component
normal
carcinoma
sarcoma
undifferentiated
mixte
methylation level
2
1
0
−1
−2
SARCOMA HYPER-METHYLATED GENES
ASSOCIATED PATHWAYS
no significantly enriched pathway
CARCINOMA HYPER-METHYLATED GENES
ASSOCIATED-PATHWAYS (FDR p-value top 20)
0.05
1e-2
REACTOME_MOLECULES_ASSOCIATED_WITH_ELASTIC_FIBRES
REACTOME_ELASTIC_FIBRE_FORMATION
MIKKELSEN_NPC_LCP_WITH_H3K4ME3
SABATES_COLORECTAL_ADENOMA_DN
MIKKELSEN_MEF_LCP_WITH_H3K4ME3
MIKKELSEN_ES_LCP_WITH_H3K4ME3
MIKKELSEN_IPS_LCP_WITH_H3K4ME3
DAVICIONI_MOLECULAR_ARMS_VS_ERMS_DN
BERTUCCI_INVASIVE_CARCINOMA_DUCTAL_VS_LOBULAR_DN
NABA_ECM_GLYCOPROTEINS
extracellular matrix
0
2
4
-log10 (FDR p-value)

Supplementary Figure 11. Differential methylation analysis (C *vs* S). Heatmap and pathway over-representation analysis of differentially methylated promoters between carcinomatous and sarcomatous (or undifferentiated) components of the ten samples derived from the five selected CS tumors. Methylation level: scaled beta-values. Gene clustering method: Ward’s; distance: Spearman. FDR: false discovery rate.
