# Supplementary material for: Dissecting the Origin of Heterogeneity in Uterine and Ovarian Carcinosarcomas
Source: Cancer Res Commun. 2023 May 10;3(5):830–41. doi: 10.1158/2767-9764.CRC-22-0520 (PMC10171113; doi:10.1158/2767-9764.CRC-22-0520)
Supplement: Figure S12 — Complete clonal evolution of P02 tumor. [file crc-22-0520-s15.pdf]

Figure S12

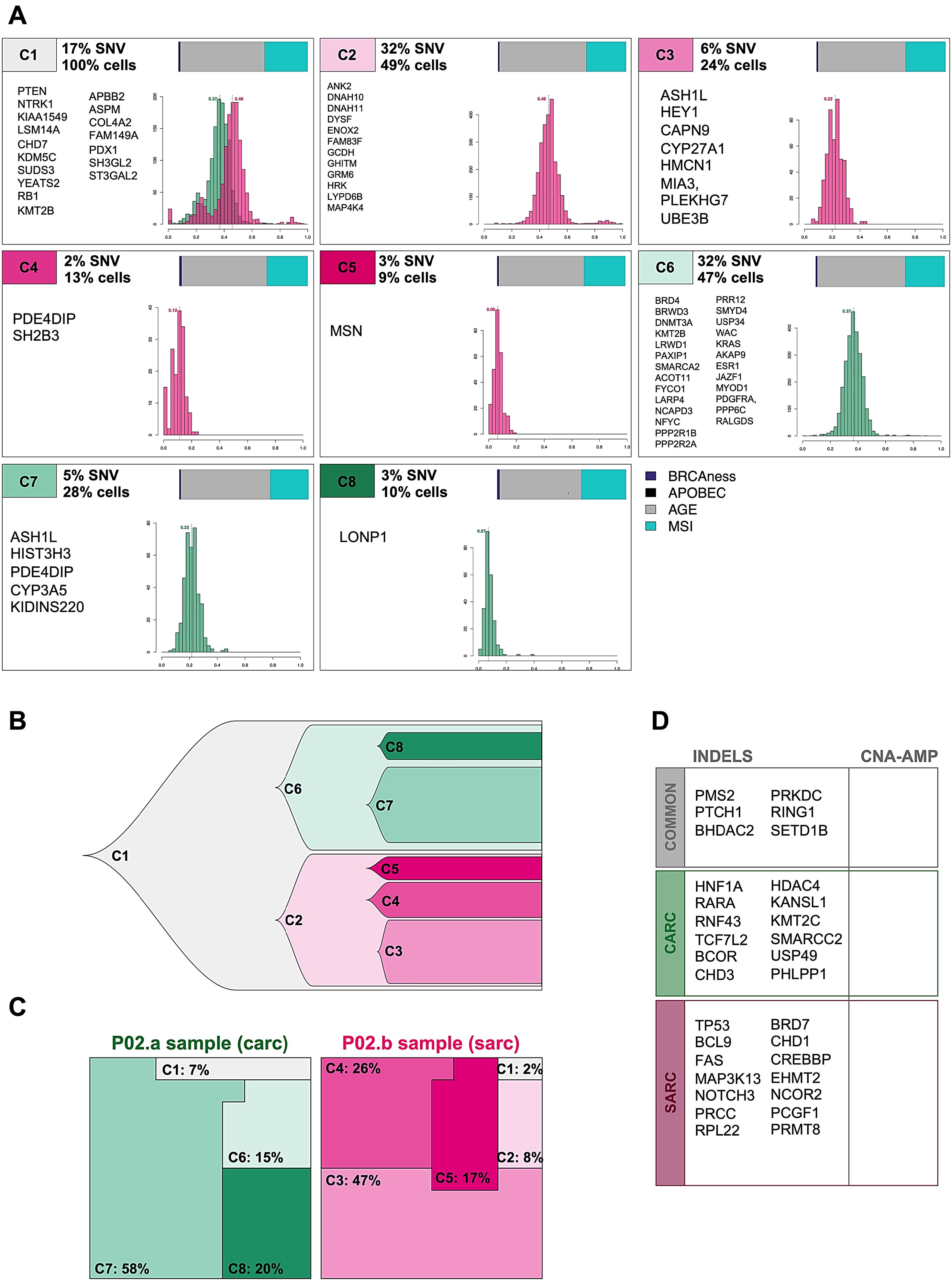

A
C1
17% SNV
100% cells
PTEN
NTRK1
KIAA1549
LSM14A
CHD7
KDM5C
SUDS3
YEATS2
RB1
KMT2B
APBB2
ASPM
COL4A2
FAM149A
PDX1
SH3GL2
ST3GAL2
0.37
0.46
C2
32% SNV
49% cells
ANK2
DNAH10
DNAH11
DYSF
ENOX2
FAM83F
GCDH
GHITM
GRM6
HRK
LYPD6B
MAP4K4
0.46
C3
6% SNV
24% cells
ASH1L
HEY1
CAPN9
CYP27A1
HMCN1
MIA3,
PLEKHG7
UBE3B
0.22
C4
2% SNV
13% cells
PDE4DIP
SH2B3
0.12
C5
3% SNV
9% cells
MSN
0.06
C6
32% SNV
47% cells
BRD4
BRWD3
DNMT3A
KMT2B
LRWD1
PAXIP1
SMARCA2
ACOT11
FYCO1
LARP4
NCAPD3
NFYC
PPP2R1B
PPP2R2A
PRR12
SMYD4
USP34
WAC
KRAS
AKAP9
ESR1
JAZF1
MYOD1
PDGFRA,
PPP6C
RALGDS
0.37
C7
5% SNV
28% cells
ASH1L
HIST3H3
PDE4DIP
CYP3A5
KIDINS220
0.22
C8
3% SNV
10% cells
LONP1
0.07
BRCAness
APOBEC
AGE
MSI
B
C1
C6
C8
C7
C2
C5
C4
C3
C
P02.a sample (carc)
C1: 7%
C6: 15%
C7: 58%
C8: 20%
P02.b sample (sarc)
C4: 26%
C1: 2%
C2: 8%
C3: 47%
C5: 17%
D
INDELS
CNA-AMP
COMMON
PMS2
PTCH1
BHDAC2
PRKDC
RING1
SETD1B
CARC
HNF1A
RARA
RNF43
TCF7L2
BCOR
CHD3
HDAC4
KANSL1
KMT2C
SMARCC2
USP49
PHLPP1
SARC
TP53
BCL9
FAS
MAP3K13
NOTCH3
PRCC
RPL22
BRD7
CHD1
CREBBP
EHMT2
NCOR2
PCGF1
PRMT8

Supplementary Figure 12. Complete clonal evolution of P02 tumor. A, Description of SNV associated with each clone detected by phyloWGS. The percentage of SNV specific to each population (from C1 to C8) among all detected SNV is indicated together with the proportion of cells relative to each clonal population. Histograms represent the VAF of SNV sets (specific to each clonal population) in the sample where they are detected (green bars for carcinoma and pink bars for sarcoma sample). The contribution of each of the four mutational signatures in each clone is estimated (horizontal bars). Mutated genes of interest in each population are listed. **B**, Clonal lineage inference. **C**, Clonal population frequencies for both tumor samples. **D**, Other gene alterations of interest (indel and amplifications) classified as common in both or specific to either carcinoma or sarcoma sample.
