# Supplementary material for: Dissecting the Origin of Heterogeneity in Uterine and Ovarian Carcinosarcomas
Source: Cancer Res Commun. 2023 May 10;3(5):830–41. doi: 10.1158/2767-9764.CRC-22-0520 (PMC10171113; doi:10.1158/2767-9764.CRC-22-0520)
Supplement: Figure S13 — Complete clonal evolution of P01 tumor. [file crc-22-0520-s16.pdf]

Figure S13

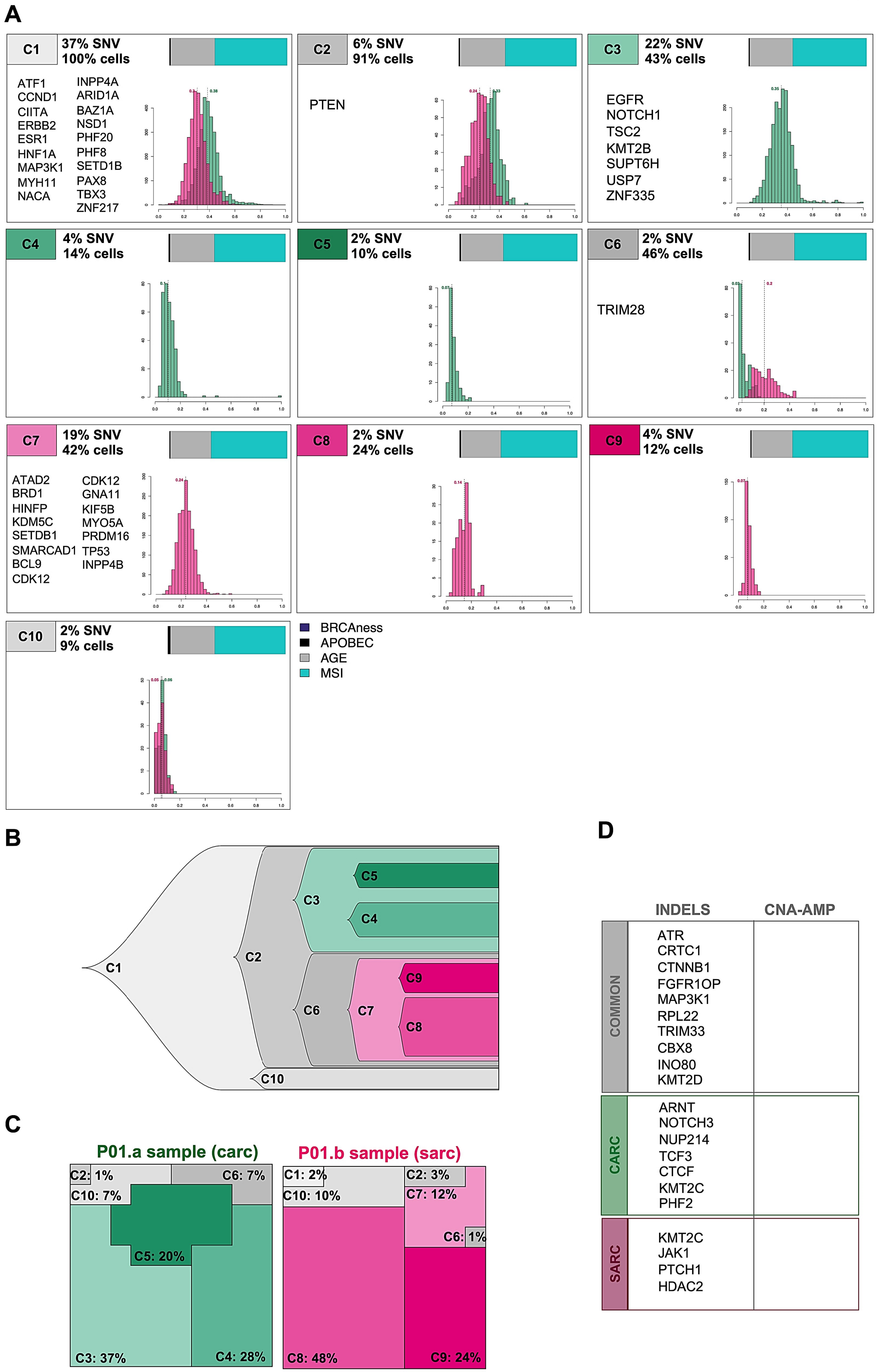

A
C1
37% SNV
100% cells
ATF1
CCND1
CIITA
ERBB2
ESR1
HNF1A
MAP3K1
MYH11
NACA
INPP4A
ARID1A
BAZ1A
NSD1
PHF20
PHF8
SETD1B
PAX8
TBX3
ZNF217
C2
6% SNV
91% cells
PTEN
C3
22% SNV
43% cells
EGFR
NOTCH1
TSC2
KMT2B
SUPT6H
USP7
ZNF335
C4
4% SNV
14% cells
C5
2% SNV
10% cells
C6
2% SNV
46% cells
TRIM28
C7
19% SNV
42% cells
ATAD2
BRD1
HINFP
KDM5C
SETDB1
SMARCAD1
BCL9
CDK12
CDK12
GNA11
KIF5B
MYO5A
PRDM16
TP53
INPP4B
C8
2% SNV
24% cells
C9
4% SNV
12% cells
C10
2% SNV
9% cells
BRCAness
APOBEC
AGE
MSI
B
C1
C2
C3
C4
C5
C6
C7
C8
C9
C10
C
P01.a sample (carc)
C2: 1%
C6: 7%
C10: 7%
C5: 20%
C3: 37%
C4: 28%
P01.b sample (sarc)
C1: 2%
C2: 3%
C10: 10%
C7: 12%
C6: 1%
C8: 48%
C9: 24%
D
INDELS
CNA-AMP
COMMON
ATR
CRTC1
CTNNB1
FGFR1OP
MAP3K1
RPL22
TRIM33
CBX8
INO80
KMT2D
CARC
ARNT
NOTCH3
NUP214
TCF3
CTCF
KMT2C
PHF2
SARC
KMT2C
JAK1
PTCH1
HDAC2

Supplementary Figure 13. Complete clonal evolution of P01 tumor. A, Description of SNV associated with each clone detected by phyloWGS. The percentage of SNV specific to each population (from C1 to C10) among all detected SNV is indicated together with the proportion of cells relative to each clonal population. Histograms represent the VAF of SNV sets (specific to each clonal population) in the sample where they are detected (green bars for carcinoma and pink bars for sarcoma sample). The contribution of each of the four mutational signatures in each clone is estimated (horizontal bars). Mutated genes of interest in each population are listed. **B**, Clonal lineage inference. **C**, Clonal population frequencies for both tumor samples. **D** Other gene alterations of interest (indel and amplifications) classified as common in both or specific to either carcinoma or sarcoma sample.
