# Supplementary material for: Dissecting the Origin of Heterogeneity in Uterine and Ovarian Carcinosarcomas
Source: Cancer Res Commun. 2023 May 10;3(5):830–41. doi: 10.1158/2767-9764.CRC-22-0520 (PMC10171113; doi:10.1158/2767-9764.CRC-22-0520)
Supplement: Figure S14 — Complete clonal evolution of P05 tumor. [file crc-22-0520-s17.pdf]

Figure S14

A

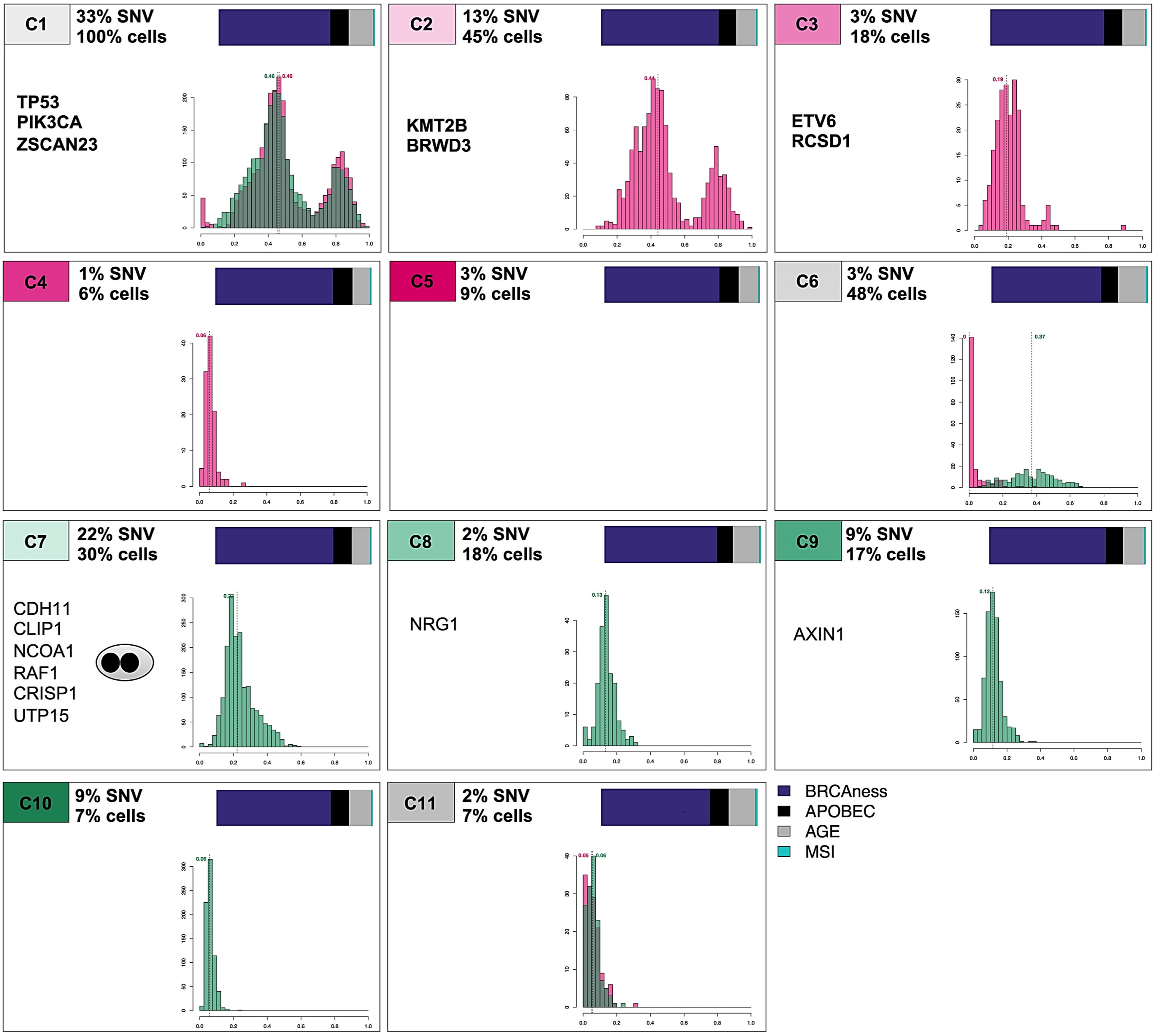

B

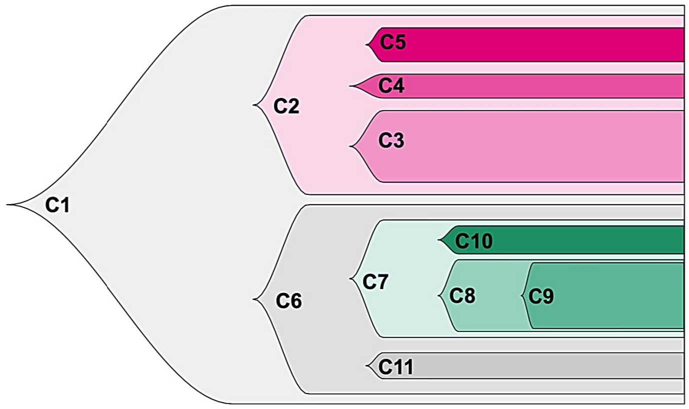

B

	INDELS	CNA-AMP
COMMON	BRCA1	
CARC		PIK3CA<br>ALK<br>MAP3K13<br>MDM4<br>SOX2<br>ACTL6A<br>DPY30<br>ING1<br>RBBP5<br>TBL1XR1<br>YEATS2
SARC		

C

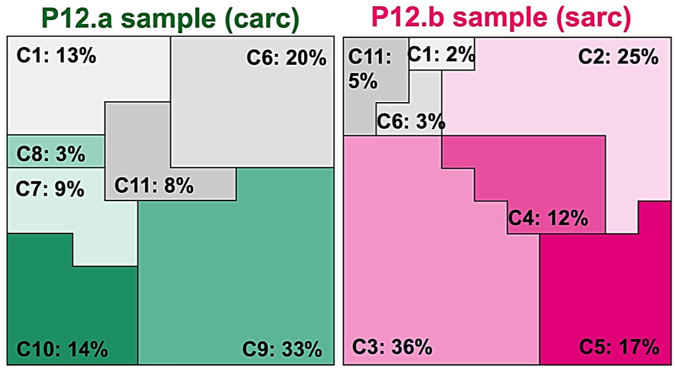

Supplementary Figure 14. Complete clonal evolution of P05 tumor. A, Description of SNV associated with each clone detected by phyloWGS. The percentage of SNV specific to each population (from C1 to C11) among all detected SNV is indicated together with the proportion of cells relative to each clonal population. Histograms represent the VAF of SNV sets (specific to each clonal population) in the sample where they are detected (green bars for carcinoma and pink bars for sarcoma sample). The contribution of each of the four mutational signatures in each clone is estimated (horizontal bars). Mutated genes of interest in each population are listed. Genome doubling process is indicated. Genome doubling process is indicated.**B**, Clonal lineage inference. **C**, Clonal population frequencies for both tumor samples. **D**, Other gene alterations of interest (indel and amplifications) classified as common in both or specific to either carcinoma or sarcoma sample.
