# Supplementary material for: Dissecting the Origin of Heterogeneity in Uterine and Ovarian Carcinosarcomas
Source: Cancer Res Commun. 2023 May 10;3(5):830–41. doi: 10.1158/2767-9764.CRC-22-0520 (PMC10171113; doi:10.1158/2767-9764.CRC-22-0520)
Supplement: Figure S15 — Complete clonal evolution of P12 tumor. [file crc-22-0520-s18.pdf]

Figure S15

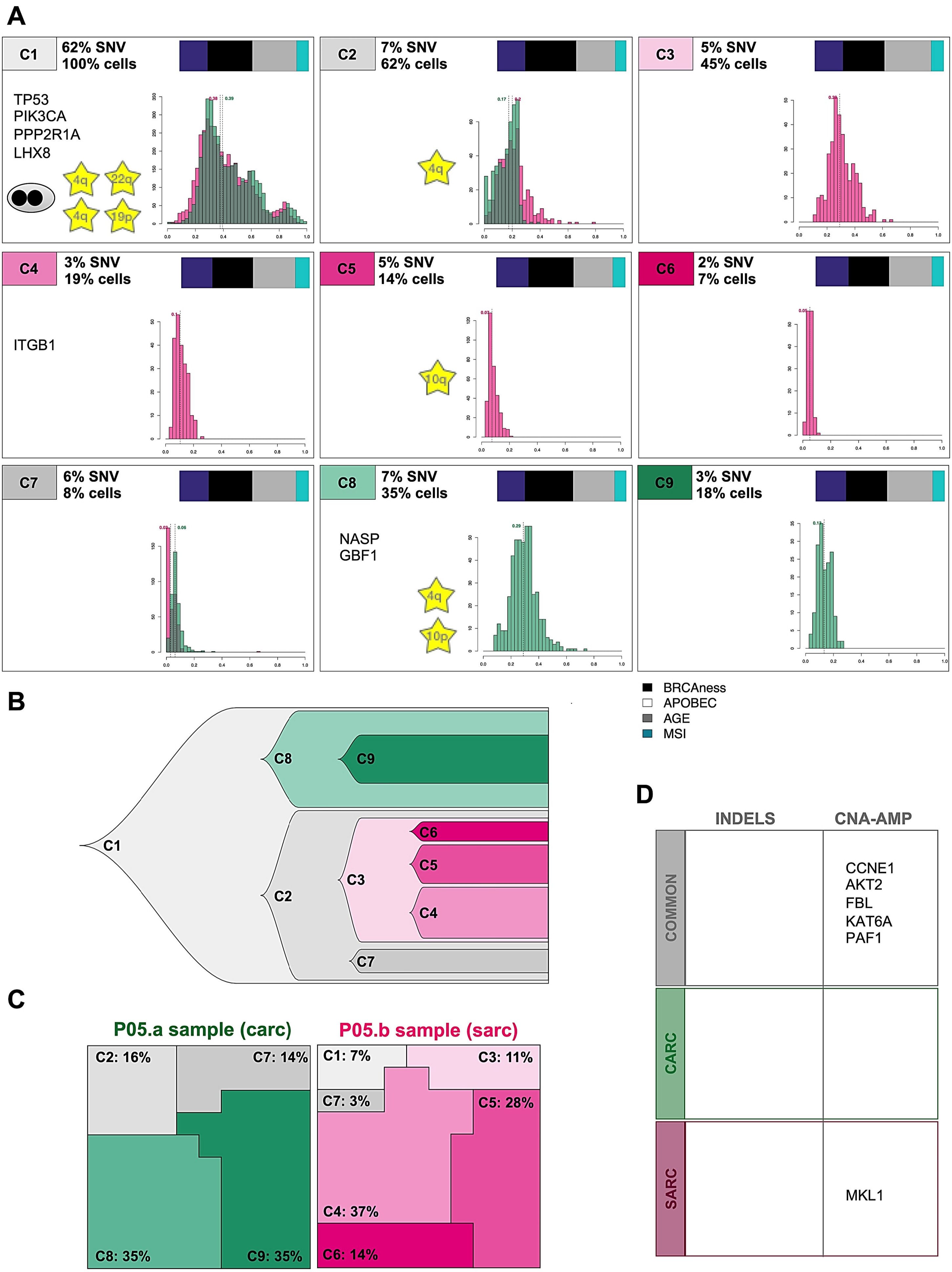
A
C1
62% SNV
100% cells
TP53
PIK3CA
PPP2R1A
LHX8
4q
22q
4q
19p
C2
7% SNV
62% cells
4q
C3
5% SNV
45% cells
C4
3% SNV
19% cells
ITGB1
C5
5% SNV
14% cells
10q
C6
2% SNV
7% cells
C7
6% SNV
8% cells
C8
7% SNV
35% cells
NASP
GBF1
4q
10p
C9
3% SNV
18% cells
BRCAness
APOBEC
AGE
MSI
B
C1
C2
C3
C4
C5
C6
C7
C8
C9
C
P05.a sample (carc)
C2: 16%
C7: 14%
C8: 35%
C9: 35%
P05.b sample (sarc)
C1: 7%
C3: 11%
C7: 3%
C5: 28%
C4: 37%
C6: 14%
D
INDELS
CNA-AMP
COMMON
CCNE1
AKT2
FBL
KAT6A
PAF1
CARC
SARC
MKL1

Supplementary Figure 15. Complete clonal evolution of P12 tumor. A, Description of SNV associated with each clone detected by phyloWGS. The percentage of SNV specific to each population (from C1 to C9) among all detected SNV is indicated together with the proportion of cells relative to each clonal population. Histograms represent the VAF of SNV sets (specific to each clonal population) in the sample where they are detected (green bars for carcinoma and pink bars for sarcoma sample). The contribution of each of the four mutational signatures in each clone is estimated (horizontal bars). Mutated genes of interest in each population are listed. Genome doubling process is indicated. Yellow stars represent chromosomal localization of kataegis events. **B**, Clonal lineage inference. **C**, Clonal population frequencies for both tumor samples. **D**, Other gene alterations of interest (indel and amplifications) classified as common in both or specific to either carcinoma or sarcoma sample.
