# Supplementary material for: Dissecting the Origin of Heterogeneity in Uterine and Ovarian Carcinosarcomas
Source: Cancer Res Commun. 2023 May 10;3(5):830–41. doi: 10.1158/2767-9764.CRC-22-0520 (PMC10171113; doi:10.1158/2767-9764.CRC-22-0520)
Supplement: Figure S16 — Complete clonal evolution of P15 tumor. [file crc-22-0520-s19.pdf]

Figure S16

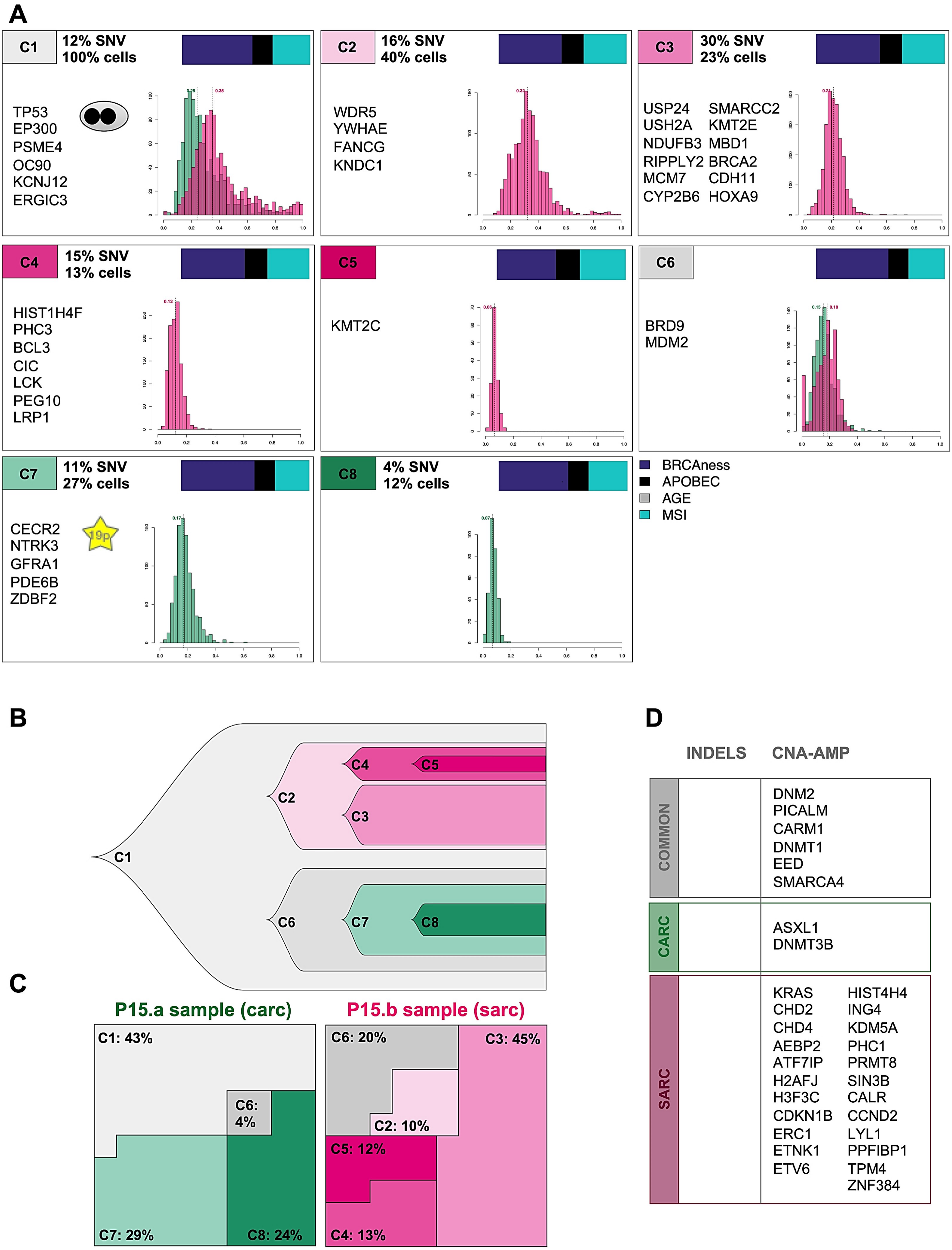
A
C1
12% SNV
100% cells
TP53
EP300
PSME4
OC90
KCNJ12
ERGIC3
C2
16% SNV
40% cells
WDR5
YWHAE
FANCG
KNDC1
C3
30% SNV
23% cells
USP24 SMARCC2
USH2A KMT2E
NDUFB3 MBD1
RIPPLY2 BRCA2
MCM7 CDH11
CYP2B6 HOXA9
C4
15% SNV
13% cells
HIST1H4F
PHC3
BCL3
CIC
LCK
PEG10
LRP1
C5
KMT2C
C6
BRD9
MDM2
C7
11% SNV
27% cells
CECR2
NTRK3
GFRA1
PDE6B
ZDBF2
19p
C8
4% SNV
12% cells
BRCAness
APOBEC
AGE
MSI
B
C1
C2
C3
C4
C5
C6
C7
C8
C
P15.a sample (carc)
C1: 43%
C6: 4%
C7: 29%
C8: 24%
P15.b sample (sarc)
C6: 20%
C3: 45%
C2: 10%
C5: 12%
C4: 13%
D
INDELS
CNA-AMP
COMMON
DNM2
PICALM
CARM1
DNMT1
EED
SMARCA4
CARC
ASXL1
DNMT3B
SARC
KRAS HIST4H4
CHD2 ING4
CHD4 KDM5A
AEBP2 PHC1
ATF7IP PRMT8
H2AFJ SIN3B
H3F3C CALR
CDKN1B CCND2
ERC1 LYL1
ETNK1 PPFIBP1
ETV6 TPM4
ZNF384

Supplementary Figure 16. Complete clonal evolution of P15 tumor. A, Description of SNV associated with each clone detected by phyloWGS. The percentage of SNV specific to each population (from C1 to C8) among all detected SNV is indicated together with the proportion of cells relative to each clonal population. Histograms represent the VAF of SNV sets (specific to each clonal population) in the sample where they are detected (green bars for carcinoma and pink bars for sarcoma sample). The contribution of each of the four mutational signatures in each clone is estimated (horizontal bars). Mutated genes of interest in each population are listed. Genome doubling process is indicated. Yellow star represents chromosomal localization of kataegis events. **B**, Clonal lineage inference. **C**, Clonal population frequencies for both tumor samples. **D**, Other gene alterations of interest (indel and amplifications) classified as common in both or specific to either carcinoma or sarcoma sample.
